# Developing intervention fidelity strategies for a behaviour change intervention delivered in primary care dental practices: the RETURN fidelity strategy

**DOI:** 10.1186/s12875-025-02732-1

**Published:** 2025-02-17

**Authors:** Victoria Lowers, B. Young, R. V. Harris

**Affiliations:** Department of Public Health, Policy and Systems, Institute of Population Health, University of Liverpool, Whelan Building, Liverpool, L69 3GL UK

**Keywords:** Fidelity, Behaviour change, Primary dental care, Intervention implementation, Dental teams

## Abstract

**Background:**

Behaviour change interventions delivered in real-world settings could be vulnerable to threats to internal and external validity if methodological considerations are overlooked. The primary dental care setting is a difficult environment within which to deliver research protocols presenting unique challenges for robust scientific research delivery. Intervention fidelity strategies are an important mechanism to improve the scientific rigor of such studies. Feasibility studies provide a vital opportunity to refine and optimise research processes and implementation strategies before embarking on larger scale projects. This paper sets out the development of a comprehensive intervention fidelity strategy guided by The National Institutes of Health Behavior Change Consortium.(BCC) recommendations.

**Method:**

Using observations (53 h) and qualitative interviews (17 patient interviews and 2 staff interviews) conducted during the delivery of the RETURN feasibility study (InteRventions to rEduce inequaliTies in the Uptake of Routine deNtal care), an intervention fidelity strategy was developed for use in the RETURN main trial.

**Results:**

A comprehensive intervention fidelity strategy was developed, structured around the five domains of the BCC’s recommendations (design, training, delivery, receipt, enactment) and attending to the goals pertaining to each of those domains. This paper sets out the fidelity strategy implemented in the RETURN main trial.

**Implications and conclusions:**

The RETURN fidelity strategy was influenced by the unique research environment the primary dental care setting presents. The strategy could serve as a blueprint to other researchers conducting research in similar settings. It is also intended that this strategy is read alongside the RETURN results upon their publication.

**Trial registration:**

ISRCTN10853330, registered: 07/10/2019.

**Supplementary Information:**

The online version contains supplementary material available at 10.1186/s12875-025-02732-1.

## Introduction

Monitoring the implementation of behaviour change interventions (BCIs) according to their intended protocols is essential for the accurate interpretation of healthcare trial results [1]. Failure to prevent unintended deviations from BCI protocols increases the risk of methodological errors, leading to uncertainties in the interpretation of results [2]. Specifically, poor monitoring of BCI implementation can result in Type I errors, where trial results falsely indicate an intervention’s effectiveness due to unauthorised additions or omissions of key components. Type II errors occur when a genuine effect is not detected for similar reasons, and Type III errors arise when incorrect conclusions about BCI effectiveness are drawn due to discrepancies between the intended and delivered interventions [3].

To mitigate unintended outcomes in trials testing BCIs, it is crucial to implement strategies that enhance both internal and external validity [4]. This ensures that conclusions drawn are unequivocally attributable to the BCI rather than extraneous factors [5]. The complexity and multi-component nature of BCIs present specific challenges in achieving scientific rigour, as isolating the effect of each component and ensuring consistent implementation across various contexts is difficult [6]. Intervention fidelity, also known as treatment fidelity, is a critical methodological tool in addressing these challenges within randomised controlled trials (RCTs) [7].

One conceptualisation of intervention fidelity tailored to BCIs is the National Institutes of Health Behavior Change Consortium (BCC) treatment fidelity framework [1, 8]. The BCC defines intervention fidelity as “the methodological strategies and practices used to enhance and monitor the reliability and validity of behavioural interventions” [1]. The framework provides a structured set of strategies for researchers to enhance fidelity practices during the development and testing of BCIs, particularly when these interventions are delivered in real-world environments by healthcare professionals [1, 8]. This approach ensures consistent and effective implementation across different settings. The BCC fidelity framework encompasses five domains: design, training, delivery, receipt, and enactment, with each domain offering specific strategies to enhance fidelity within that area [1]. Table 1 provides an overview of how the strategies recommended by the BCC enhance intervention fidelity within each domain.

**Table 1:** NIH BCC intervention fidelity domains and strategy aims [1]

NIH BCC Fidelity Domain	Domain specific strategy aims	Strategy target
Study design	Strategies to ensure that a behaviour change study adequately tests it hypotheses in line with its underlying theory and / or clinical guidelines	Interventionist
Training	Strategies to standardise (or individualise where required) training to ensure that all trainees have been satisfactorily trained to deliver the behaviour change intervention consistently and competently	Interventionist
Delivery	Strategies to monitor and improve the delivery of a behaviour change intervention through its standardisation to ensure the intervention is delivered as it was intended	Interventionist
Receipt	Strategies to monitor and improve the ability of patients to perform the intervention skills provided through intervention delivery	Patient
Enactment	Strategies to monitor and improve the ability of patients to perform the intervention skills provided through delivery in real-life settings	Patient

The recent iteration of the Medical Research Council’s (MRC) guidance on the development and evaluation of complex interventions underscores the necessity for a flexible, iterative, and context-dependent approach to ensure that research findings are applicable and beneficial to real-world settings [9]. The framework specifically emphasises the value of transitioning between research phases, allowing for the integration of new insights and the refinement of interventions to optimise outcomes [10]. Feasibility studies play a crucial role in this iterative process, enabling researchers to identify and address potential implementation challenges, assess recruitment and retention rates, and test the practicality of procedures on a smaller scale before advancing to full-scale evaluations [11]. Accordingly, it is essential that the refinement of intervention fidelity strategies is embedded within the outcomes of feasibility studies, with a particular focus on context-dependent factors and the incorporation of relevant stakeholder perspectives [9].

The primary dental care setting poses distinct challenges for implementing BCI trials, and dental trials often lack methodological rigor, resulting in ambiguous findings [12]. Several factors contribute to these challenges. The decentralised nature of primary care dental practices, which often function as independent business entities, complicates the standardisation and coordination necessary across multiple study sites [13]. Additionally, the significant variability in patient populations within dental practices hampers the development of uniform research protocols [13]. The busy and high-demand environment of dental practices also restricts the time available for dental teams to participate in research activities [14], thereby affecting the feasibility of conducting methodologically rigorous studies. Whilst many of these challenges could apply to other primary care settings (i.e. General Practice), the primary dental care setting within the United Kingdom (UK) is also an untapped research setting [15] suggesting dental personnel are relatively inexperienced in research delivery further adding to the challenges of delivering robust research.

To address these issues, enhancing intervention fidelity strategies in trials testing BCIs within primary dental care settings may be a viable solution. However, a recent scoping review revealed that little emphasis has been placed on the development and implementation of robust fidelity strategies in this field to date [16]. Implementing these strategies could potentially improve the reliability and consistency of outcomes in BCI trials conducted in primary dental care settings.

Accordingly, this paper aims to describe the development of a comprehensive intervention fidelity strategy for implementation in a RCT (inteRventions to rEduce inequaliTies in the Uptake of Routine deNtal care RCT – the RETURN main trial) which assesses a BCI delivered within primary dental care settings. Drawing upon principles outlined in the BCC framework, the strategy’s development has been informed by insights gathered from the RETURN feasibility study.

## Methods

### Ethics

Ethical approval was obtained from Bromley Research Ethics Committee (19/LO/1510). Research governance approvals were obtained from the Health Research Authority (reference 265789), and sponsorship was provided by the University of Liverpool (reference UoL001354). All data used were accessed only by authorised study members and were stored in a secure location in accordance with ethical requirements.

### Procedures

#### The RETURN intervention

The RETURN intervention is a single-session, brief psychological intervention delivered by dental nurses in urgent dental care settings. Its primary objective is to support patients who only use dental services when they have an urgent problem. By assisting patients in identifying and overcoming barriers to routine dental visits, the intervention aims to promote regular, planned dental care, thereby improving oral health outcomes.

The intervention is multifaceted and comprises several components delivered opportunistically to patients attending an urgent dental appointment. It leverages a “teachable moment” approach [17]. A comprehensive description of the intervention has been detailed elsewhere [18]. Briefly the intervention comprises two elements:

1. A “patient pack” with behaviour change techniques embedded within the materials. The pack comprises:

-  Six booklets addressing common barriers to routine dental visiting (cost, time constraints, not thinking to go when not in pain, distrust of dentists, embarrassment, and anxiety).
-  Corresponding barrier videos featuring dental patients sharing their experiences of overcoming barriers, augmented with engaging animations. These videos were created specifically to resonate with the trial population.
-  A written goal and action plan completed during the intervention session, targeting one barrier.
-  Additional materials and booklets intended for post-appointment use at home, encouraging routine dental visiting. These include practical aids such as breathing exercises for anxious patients, contact information for dental services, and an “employer card” endorsing routine dental attendance.
-  Access to a study website via a personal login where all intervention materials can be viewed.

2) A structured conversation facilitated by trained dental nurses [19]. The conversation guides patients through the intervention process, utilising empathetic listening, non-judgmental, and non-directive dialogue. Its dual purpose is to enhance participant engagement by tailoring discussions to individual experiences whilst ensuring interaction with key intervention components.

#### The RETURN feasibility study

The RETURN feasibility study was a parallel group, two-arm, RCT that aimed to recruit 60 patients. Its primary objective was to assess the feasibility of conducting a larger RCT (the RETURN main trial) within urgent dental care settings to evaluate the RETURN intervention. Patients were allocated to either receive no intervention (usual care at the recruiting urgent dental care site) or the RETURN intervention.

The study was conducted in Merseyside, North-West England in the UK in three site types: (1) an urgent clinic in a Teaching Dental Hospital (2), an out-of-hours urgent dental care service, and (3) an urgent clinic in an in-hours dental practice. Each site put forward dental nurses to be trained for one hour in Good Clinical Practice principles [20] and for two hours in study procedures and intervention delivery. Training sessions were didactic, with opportunities to role play intervention deliveries and were conducted either at the University of Liverpool or at site. During the recruitment period (January 2020 to March 2020), routine dental care appointments for new National Health Service (NHS) patients were readily available in the region.

Recruitment ceased abruptly due to the COVID-19 pandemic, resulting in the enrolment of 28 patients, approximately halfway to the target. Follow-up was achieved for 82% of the patients via telephone, email, or post, four months post-recruitment.

Feasibility measures included primary outcome data completion rates, recruitment rates, and fidelity. A comprehensive study description and results have been published elsewhere [21]. Briefly, despite premature termination, the results were considered sufficient to warrant proceeding to a full-scale RCT, with the addition of an internal pilot to monitor progress.

#### Developing the fidelity strategies

In alignment with MRC guidance on intervention refinement [9] and Borrelli’s [8] recommendation to pilot test interventions and incorporate feedback from participants and providers, the RETURN feasibility study provided an opportunity to develop fidelity strategies for the RETURN main trial. A total of 58 h of observations were conducted, covering the recruitment of 24 patients and 11 intervention delivery sessions (the remaining 13 patients were allocated to the control arm, and therefore, no intervention delivery was observed). Observation time also encompassed site set-up, informal discussions with dental nurses, and additional ad hoc training conversations throughout the study period. Field notes were recorded at the end of each day. Additionally, telephone semi-structured interviews were conducted with two dental nurses involved in delivering the feasibility study and 17 study patients. All dental nurses who were both trained and delivered the intervention were invited to be interviewed. This meant that numbers of dental nurses participating in interviews were limited because the onset of the COVID pandemic meant a refocusing of the dental workforce on purely clinical activity and so fewer nurses (*n* = 2) delivered the intervention than were trained (*n* = 9). This also resulted in a researcher recruiting patients at one site instead of dental nurses, but they were not approached to take part in this study. For pragmatic reasons, the 17 patients who responded to the RETURN feasibility study follow-up by telephone were invited to be interviewed (*n* = 9 intervention & *n* = 8 control), all of whom agreed to take part. Interviews were audio-recorded and transcribed.

Employing the Framework Method [22] guided by the BCC recommendations [1] and using a deductive approach [23] to structure the coding framework in accordance with the components of the intervention, field notes and interview transcripts were analysed to pinpoint areas where fidelity could be strengthened for the RETURN man trial. Data underwent coding, charting, mapping, and verification across the entire dataset to inform the development of a robust fidelity strategy.

In addition to the logic model which sets out the underlying mechanisms of the intervention materials as described in the intervention development publication [18], and recognising the two separate elements of the intervention (the “patient pack” and conversational element), we have produced an ‘operational model’ [24], to facilitate a complete assessment of intervention fidelity within the RETURN main trial [25]. This can be found at Table 2. This operational model provides a scaffold for the intervention fidelity strategy and helped inform its design, by outlining which intervention activities should be present in an intervention conversation for it to be considered delivered as intended. From this model, many of the strategies contained in this manuscript were developed (for example, it guided the components featured in the RETURN fidelity checklist developed to monitor training and assess delivery fidelity, discussed within the ‘delivery’ section below).

**Table 2:** Operational model for the RETURN intervention (post-feasibility)

Resources	Activities	Outputs	Outcomes	Impact
• Dental nurses with capacity for research activities (including training time) • Dental nurses with the competency to deliver the intervention (achieved through training) • Dental practices with eligible participants • Appropriate physical space within dental practices to deliver interventions • IT infrastructure to support the intervention delivery session (tablet PC for video viewing and Wi-Fi connection)	• 1:1 discussion with participants about barriers to dental visiting • Application of MI inspired communication style • Showing a relevant intervention barrier video • Going through a relevant intervention barrier booklet • Goal setting and action planning using SMART principles • Setting intentions for future behaviours through discussion • Provision of the “Patient pack”	• Increased motivation around routine dental visiting • Increased self-efficacy around round dental visiting • Reduction in feelings of worry around dental visiting • Increased knowledge around routine dental visiting • A tangible goal and action plan to refer to • Participants use intervention pack outside of the setting to support dental visiting	Attendance at a routine dental appointment within 18 months of the intervention delivery	• Reduction in oral health and routine dental visiting inequalities • Improved oral health outcomes

## Results

The BCC recommendations were used as a guide to develop a comprehensive fidelity strategy for the RETURN trial, addressing the lessons learned from the conduct of the feasibility study. This article now sets out the strategy following the 5 domains (**in bold**) and goals (***in bold italics***) of the BCC framework [1], adapted by Borrelli [8].

### Design

#### Explicitly identify and use a theoretical model as a basis for the intervention and ensure the intervention components and measures are reflective of underlying theory

The theoretical underpinnings guiding the intervention have been detailed in a publication outlining the intervention’s development process, which includes a comprehensive logic model [18]. Briefly, the intervention draws from multiple theoretical frameworks, incorporating elements of Protection Motivation Theory [26] and Identity-based Motivation Theory [27].

During the feasibility study, it became evident that the conversational aspect of the intervention required a structured approach to enhance standardisation across intervention sessions. Accordingly, Motivational Interviewing (MI) ‘spirit’ [28] was introduced as a framework to provide structure to these conversations in the RETURN main trial, while bolstering the theoretical coherence of intervention deliveries.

#### Ensure consistent intervention dose and develop a monitoring plan to maintain consistency

Variations in intervention ‘dose’ were noted during the feasibility study, with session durations ranging from 10 to 37 min, Mean (Standard Deviation (SD)) = 21 [8] minutes. Observations revealed this was primarily influenced by patient engagement and confidence levels of the interventionist.

Whilst an intervention duration target of around 15 min was set for pragmatic reasons as part of the intervention design goals for the feasibility study, for the RETURN main trial, a larger emphasis on dose standardisation will be implemented through training. Additionally, to underscore the importance of regulating dose, specific guidance on the duration for each intervention component for the main trial will be given:

- Barrier discussion – 4 min.
- Motivation enhancement: video and discussion – 3 min.
- Knowledge enhancement (guided discussion using booklet materials) – 3 min.
- Setting Specific, Measurable, Achievable, Relevant, and Time-bound (SMART) goals and action plan – 4 min.
- Setting intentions at the session’s conclusion – 1 min.

However, as a patient-centred approach is inherent in MI techniques where discussions are led by patients, variations in intervention dose will be deemed an acceptable intervention adaptation in the RETURN main trial. This decision is supported by the understanding that patients facing multiple barriers may have longer ‘barrier discussions’ leading to variations in intervention duration. Monitoring of dose will be achieved through audio-recordings, although no corrective measures will be taken to standardise dose in the main trial.

Patients also take intervention materials home, and accordingly, questions about additional engagement with the materials will form part of the RETURN main trial follow-ups. Likewise, metadata from the study website will be reviewed to assess whether patients viewed intervention materials at home. This comprehensive approach to dose monitoring aims to enrich the interpretation of the RETURN main trial findings, and dose variations will be considered in the analysis of study outcomes (i.e. is there an optimum amount of ‘dose’ to elucidate behaviour change? ).

#### Develop a plan for how adherence to the protocol will be monitored. Monitor both intervention delivery and assessment administration

Adherence to the intervention protocol was identified as a concern during the feasibility study. Based on observations, only 5/11 (45%) intervention patients received the intended discussion. The following feasibility observation illustrates poor adherence to the prescribed approach:

DN02: “*This is the pack; they have spoken to lots of people to make the pack. There are 6 barriers that people told to them. These are common and lots of people said them*”.

Observation: DN02 was showing the ‘What Next Booklet’ to the participant but kept it in front of them so the participant was unable to read it. DN02 flashed the booklet and pointed to the barriers. Moving the booklet away again, they read the barriers out one by one.

DA0201: “*So we have cost*,* time*,* I don’t think I have any problems*,* trust*,* embarrassment*,* anxiety”.* The nurse turned the booklet over, and said *“and there is also a plan*,* that is from psychological theory*,* and there are other materials*”. All the while DA02 kept hold of the booklet.

Observation: DN02 then went back to the barrier page, showing the participant and asked: “*Which of these do you relate to?*” I felt this was quite a closed question. There was no conversation about what was stopping them from going. The participant was simply asked to choose which one from the list.

Observation 06: Site 02, DN02.

To address this in the RETURN main trial, adherence monitoring will be strengthened by considering the challenges of the research context. Indeed, findings from a recent scoping review of fidelity reporting in primary care dental settings [16] suggests the onus/burden of intervention protocol adherence and competency monitoring should sit with research teams. Therefore, in the RETURN main trial, dental nurses will be asked to audio-record 100% of their intervention sessions, rather than alternative monitoring techniques such as asking them to complete checklists after each intervention delivery.

Adherence and competency during the RETURN main trial will be monitored by selecting at least one intervention recording per dental nurse each month which the research team will score using pre-determined criteria contained within an intervention specific fidelity checklist (the RETURN checklist, see Table 3). The RETURN checklist comprises 6 essential intervention components: overarching communication skills (MI derived), barrier discussion, motivation enhancement through a video, knowledge enhancement through a barrier booklet, goal and action plan setting, intention setting. Each component comprises a combination of theoretical components designed to increase behaviour change capacity (i.e. encouraging the use of SMART principles for goal setting) and practical requirements (i.e. showing the video relevant to the selected barrier). The scoring system takes the form of a Likert-scale: 0 = not implemented, 1 = partially implemented, 2 = substantially implemented, 3 = fully implemented, to give an indication of both adherence and competency.

**Table 3:** RETURN fidelity checklist

RETURN fidelity checklist Dental Nurse Code:		Fully Implemented Score: 3	Substantially Implemented Score: 2	Partially Implemented Score: 1	Not implemented Score: 0
**Overarching communication skills**					
Use of empathic listening statements					
Use of relevant open questions					
Use of non-judgemental language					
Use of non-directive talk					
Patient’s priorities, beliefs and challenges acknowledged					
**1: Discuss barriers to regular dental attendance – raise awareness**					
Patient given space to tell their story (if they choose to do so)					
Patient encouraged to come up with their own barriers without being led, or spoken for					
Patient encouraged to decide on the one barrier they want to work on					
**2: Increase Motivation**					
Patient is shown the video relevant to their selected barrier					
Encouragement provided to the patient to reflect on the video, and how their own situation relates					
**3: Increase knowledge**					
Patient guided to the booklet relevant to their selected barrier					
Information (relevant to the barrier) provided to the patient					
Statements communicated offering hope and assurances to the patients about their ability to overcome barriers					
Emphasis placed on the benefits of regular dental attendance					
**4: Setting SMART goals and action plans**					
SMART principles applied to goal and action plan					
Patient guided to set their *own* goal and action plan tailored to their situation					
Photographs of goal and action plan taken					
**5: Increase Intention**					
Encouragement statements about what’s been achieved in the session					
Encouragement provided to the patient to look at intervention materials at home					
**Feedback**:					
**Strengths**					
**Areas for development**	Unhelpful components present in session: □ Providing directive clinical advice □ Telling the patient what they should or should do / have done □ Setting goal / action plan for the patient □ Choosing the barrier for the patient				

There are no guidelines to inform the optimum ‘level’ of fidelity that should be present in a BCI delivered within dental practices. However, Durlak and DuPre found outcomes were effective in educational interventions if they were delivered with 60-80% fidelity [29], and a 90% threshold is frequently used in clinical interventions involving psychological therapies [30]. Therefore, a cautious approach will be adopted in the RETURN main trial and a threshold of 80% within each intervention component will be set for a delivery session to be considered to have achieved high fidelity.

To provide guidance and to ensure consistency in intervention scoring, a scoring guidance manual was created (see Additional File 1). This was developed collaboratively by RETURN researchers using an iterative approach to ensure that the descriptions contained within the manual were understood consistently across the team. The manual was both created and tested using a method whereby audio-recordings of the feasibility intervention sessions were scored independently, results compared, and discrepancies discussed until consensus was achieved (> 80% agreement rate).

The development of the fidelity checklist and the scoring guidance manual followed steps three to five as suggested by Walton and colleagues [31], with an iterative approach utilising feedback from the RETURN researchers to refine the items and scoring guidance.

An example of the scoring guidance for the domain of ‘overarching communication skills’ for the demonstration of ‘priorities, beliefs and challenges acknowledged’ is illustrated below:

##### Patient’s priorities, beliefs and challenges acknowledged

patients should not be challenged on their beliefs, priorities or challenges experienced previously, even if they are in direct conflict with the principles of the delivery nurse. These should simply be acknowledged as an experience that occurred.

- Score 0 if patient’s priorities/beliefs are challenged by the nurse e.g. Patient: “I couldn’t get a dentist because there weren’t any” Nurse “There was loads of NHS availability a year ago so that can’t be true”.
- Score 1 if some attempt is made to acknowledge but the patient’s priorities/beliefs are also challenged e.g. Patient “I couldn’t get a dentist because there weren’t any” Nurse “It sounds like it was really difficult for you to get yourself into the dentist, but there were dentists available”.
- Score 2 if patient’s priorities/beliefs and challenges are acknowledged during most of the session, but once or twice the nurse challenged the patients on these.
- Score 3 if acknowledgments rather than challenges are present. Patient: “I couldn’t get a dentist” Nurse: “Sounds like it was really tricky for you to get into a dentist in the past”.

Evaluation procedures to support scoring throughout the RETURN main trial will also include the consistent use of the same scoring team and the employment of interrater reliability methods. Where an agreement rate of less than 60% is found between team members responsible for scoring throughout the course of the trial, additional scoring training will take place, again using inter-rater reliability to determine agreement rates.

The RETURN checklist has been designed as a multi-functional tool for the implementation of fidelity strategies. Its functions are to act as a standardised training aide, a method to set competency expectations, a means of leveraging feedback to interventionists, a means of monitoring protocol adherence and competency levels throughout the main trial, and to assess the level of fidelity achieved in intervention deliveries at the end of the trial.

#### Develop a plan to record intervention protocol deviations and a method for providing timely feedback to interventionists

Several strategies were developed to document and address protocol deviations in the RETURN main trial:

- A coaching culture will be integrated into the training methodology to promote open communication and rapport between trainers and dental nurses. This aims to facilitate an environment where protocol deviations would be more likely to be reported, and where feedback would be enacted. This will take the form of regular, personalised, and constructive feedback designed to encourage confidence and build both communication and intervention skills.
- Additionally, monthly, each nurse will have at least one intervention audio-recording evaluated using the RETURN checklist with strengths and any areas for improvement noted. Checklists will be provided to the dental nurses once completed. Where low scores are found, additional intervention sessions will be scored, supplemented with a support site visit. Booster training will trigger where necessary through consistent low scores using the RETURN checklist.
- The protocol deviation plan will be clearly communicated to dental nurses at the outset of the RETURN main trial set-up phase. This transparent approach aims to cultivate an environment where protocol deviations are viewed as opportunities for learning rather than punitive measures.

#### Develop a user-friendly scripted intervention manual to ensure consistency of delivery and adherence to active ingredients of the treatment

Learning from the feasibility study suggested that using scripted approaches to intervention delivery were unsuccessful, as is demonstrated in the following observation:

The nurse opened the pack and put it on the table. They read through the patient pack introduction information printed on the materials very quietly, not making eye contact with the patient as they did this. The patient was listening intently, leaning forward slightly to be able to hear what the nurse was saying.

I felt some of the meaning was lost during this explanation, as the nurse was so quiet and stilted, it was difficult to hear. The nurse came across as very unconfident and reliant on the written materials. This created no room for the patient discussion.

Observation 02: Site 02, DN02.

For the RETURN main trial therefore, there will be a conscious move away from scripted materials, and instead, training intensity will be increased. In addition, an easy-to-follow intervention crib sheet was developed (see Additional File 2), alongside a written intervention training manual, designed to support intervention delivery beyond training (see Additional File 3).

#### Plan for implementation setbacks

During the feasibility study, limited resources at sites resulted in just one nurse from each of the two sites taking part in research activities, despite delivering training to multiple nurses in all three sites. As research activities were intended to integrate into nurses’ regular duties within urgent dental care settings, this constraint contributed to recruitment delays, exacerbated by factors such as COVID-19, staff sickness or holiday leave.

To address these challenges in the RETURN main trial, additional ‘float’ dental nurses will be employed as part of the core research team to carry our research duties across sites, utilising funds earmarked for reimbursing dental practices for staff time spent on research activities. Furthermore, efforts will be made to train multiple dental nurses at each site, where feasible. These strategies will form part of the early site communications.

#### Minimize contamination between conditions

Contamination was not found to be an issue within the feasibility study. Nonetheless, in the RETURN main trial, training will be provided around the importance of allocation adherence. In addition, portable research activity flow charts detailing the specific actions to follow within each study arm will be provided, supported by regular site visits from the research team.

Questions will be included in the RETURN trial follow-up pertaining to contamination (i.e. control group question: ‘Did you receive any materials at your urgent care appointment to help you to find a dentist? If so, what did that look like? ).

### Training

Training was identified as an area for improvement during the feasibility study.

#### ‘Hiring’ dental nurses to deliver the RETURN intervention

Confidence was found to be a major contributing factor to intervention delivery success, detailed in the feasibility observation below:

I passed the booklet back to the nurse, and they started going through the booklet. They didn’t explain what the booklet was for. They read out the title on each page loudly, but the rest of the information on the pages was said very quietly and sound a little muddled. The walk through of the booklet didn’t flow, and it’s more like they were reading it to them themselves under their breath to familiarise themselves with the content.

DN02: (page 2) “*For healthy teeth do I need to go?*” “*This is Megan*,* you can see about her story on the video*”. Page 3 is skipped. Page 4 “*Keeping on top of it*” “*It’s important to go all the time*” Page 5 “*This is a picture of a tooth that only the dentist could see*,* it shows the decay*”.

It is very difficult to hear what DN02 was saying, and the overall feeling is someone who lacks confidence. I felt harsh making them deliver when clearly, they didn’t feel ready with any confidence.

Observation 05, Site 01: DN02.

At the setup phase of the feasibility study, it was stipulated that effective delivery of the intervention would require experienced nurses with proficient communication skills. This expectation was based on the belief that such traits would facilitate the skills required to successfully deliver the intervention. However, implementation revealed challenges to this ideal. Informal discussions with dental staff at sites, recorded in field notes, noted that dental teams could use trial participation as an opportunity to enhance the communication skills of their staff involved in the research. This experience highlights the existence of conflicting priorities when conducting research.

As we found dental nurse attributes cannot be guaranteed, the RETURN main trial training will include elements specifically designed to increase confidence and communication skills, including enhanced role-play and a coaching style training approach. In addition, training sessions will not be fixed in length, and instead provision will be based on individualised need. This will be achievable as shadowing training is planned to occur concurrently with patient recruitment, so as not to hamper trial progress.

#### Standardise training

The BCC framework recommends training all interventionists together. In the primary dental care setting, this would require inviting dental teams to converge in a mutually convenient location, and taking staff members out of clinic was found to be problematic during the feasibility study. Instead, in the RETURN main trial, a model will be used where site personnel are trained together. As this method could affect the standardisation of the training delivered, multiple strategies were designed to mitigate that risk:

- Implementation of a ‘train the trainers’ training model led by a clinical psychologist.
- Using the same team of trainers throughout the trial.
- Using identical training materials for each site.
- Using the same role play tasks with all teams trained.
- Using a training manual and training videos.
- The development of a central website to house all training materials, as well as providing hard copies of all materials to all trainees.
- Use a training content checklist to ensure all training components were delivered to all dental teams (see Additional File 4).

#### Ensure dental nurse skill acquisition

Skill acquisition was not measured as part of the feasibility study. However, observations demonstrated variation in competency between dental nurses. Therefore, a plan was developed for the RETURN main trial to test skill acquisition during the different phases of training:

##### Training phase 1

Good Clinical Practice Training – A one-hour online module. Skill acquisition measured through an online quiz, with a pass mark of 80%.

##### Training phase 2

Intervention training – three hours, face to face delivery with a mixture of didactic learning, open discussions, and role plays. Skill acquisition measured through discussion and observations by a RETURN trainer through an intervention delivery skill acquisition checklist (see Additional File 5).

##### Training phase 3

On the job shadowing training – the amount will depend on demonstration of competencies. Skill acquisition will be measured through in vitro observations using the RETURN checklist. Each interventionist will need to achieve a score of 80% within each intervention component in a single session to be signed off as competent to deliver the intervention independently. Scoring will be conducted by the RETURN trainers and scoring decisions will be supported by the guidance manual.

#### Minimise ‘drift’ in dental nurse skills

Skills drift was not explicitly monitored during the feasibility study. However, from feasibility observations, it was discovered that intervention skills needed to be practiced regularly to be maintained. Therefore, a strategy to reduce skills drift was developed for the RETURN main trial:

- Frequent (at least one per month, per nurse) scoring and feedback of audio-recorded interventions using the scoring checklist, including elaborating strengths and areas for development.
- Triggered site visits to provide additional booster training and support in the event of low scoring (< 60% in any one component).
- Triggered (by consistent low scores) or requested reflective practice sessions, wherein a selected audio-recording will be discussed with the dental team at site, focusing on intervention elements that went well, and things that could be improved or done differently.
- Maintaining a collaborative coaching style approach to all feedback provision, booster training and reflective practice sessions to maintain relationships between the trainers and the dental nurses.

#### Accommodate dental nurse differences

Stark differences between the skills and experience levels of the feasibility dental nurses were found. The dental nurses involved in the delivery of the RETURN intervention study were not selected by the research team, they were volunteered by the dental practice owners / managers due to their availability and expression of interest in taking part. DN02 had less than 2 years’ experience of dental nursing and lacked confidence with patient communication. DN01 had more than 10 years’ experience, demonstrated good communication skills and overall was more confident in their approach to the intervention.

This quotation from DN02 describes this:

Yeah. I don’t know it might be easy for other nurses but for my range of vocabulary to like GCSE, maybe some words I found difficult, and how it works, like the way it’s [training materials] worded was difficult. If it was more informal, like ‘What are we going to do?’ ‘We’re going to do this’. Like a chatty kind of presentation maybe.

Interview with DN02

There were also differences in day-to-day responsibilities within their respective dental practices, with DN01 taking a more patient engaged role than DN02.

These contrasting quotations demonstrate this:

It’s very difficult, you know, especially for nurses because they do not have a lot of contact with patients. It’s only the dentist that takes over everything. So we do our own bit in surgery, cleaning, helping, but we don’t have conversations like that with patients.

Interview with DN02

I like talking to patients and I like the interaction and chatting with them and, you know, talking to different people as well and finding out their barriers. I think we seem a bit more human to them as well when we sit down and have a chat with them and we’re not just the scary people who work in the dentist.

Interview with DN01.

An additional challenge identified during the feasibility study was the need for training to encompass multiple methods, accommodating a wide range of baseline research skill levels. This was highlighted by the following observation on the first day of recruitment at site 02:

The nurse [DN02] told me that during the feasibility study training, they didn’t know what the word feasibility meant. They described that this word was in big letters on the very first training slide and all they could think about was wanting to Google what that word meant, so found it difficult to keep up with the rest of the training.

Observation 01, Site 02: DN02

To maintain training standardisation whilst also acknowledging the challenge of variation between nurses likely be experienced in the main trial, an ‘on-the-job shadowing’ training element was developed.

Shadowing training will involve a RETURN team member ‘chaperoning’ a dental nurse whilst they deliver interventions. Tailored support will be provided alongside real-time verbal and written feedback. This training is not time limited. Training will continue until the nurse both demonstrates competency through the scoring checklist and articulates to the trainers that they feel they have achieved a level of confidence sufficient to deliver the intervention independently. This style of ‘on-the-job’ shadowing training was developed for its ability to be highly individualised, and because it reflects the stye of training routinely undertaken by dental nurses in primary care.

#### Enhance buy-in from dental nurses

Enhancement of dental nurse buy-in was considered a priority for the upcoming RETURN trial. Within the dental practice setting, a practice owner often acts as the gatekeeper to research conduct. Those carrying out the research become involved later in the process, with vital opportunities to increase buy-in often missed. Accordingly, a series of dental nurse buy-in strategies were developed for implementation in the RETURN main trial:

- Continuing Professional Development (CPD) accreditation for all training.
- Training components designed to explain the purpose of the research, paying particular attention to patient benefit.
- An early interactive information session including dental nurses, highlighting the opportunities presented by the trial for enhanced patient interaction and training.
- Inclusion of communication skills training targeted to dental nurses.
- Monthly newsletters aimed at dental nurses and wider practice staff, with the addition of real dental nurse stories about their involvement in the trial and a quiz and prize element.
- Engagement lunches for dental nurses as a reward for participation.
- Use of communication modes congruent with dental nurse preferences i.e. WhatsApp messages rather than emails.
- Regular site visits to increase self-efficacy and confidence with research activities.
- Dental nurse awards evening to celebrate trial achievements (i.e. best recruiter etc.)

### Delivery

#### Use a scripted curriculum or treatment manual

Based on feasibility observations, scripts will not be utilised in the RETURN main trial. Instead, a selection of prompts will be provided to the nurses to ensure the intervention’s essential components are delivered. These prompts will take the form of the training manual (including intervention delivery cheat sheets), the intervention crib sheet, and videos demonstrating intervention delivery. Some components however, are ‘scripted’ within the intervention materials themselves, such as the goal and action planning section (see Additional File 6).

#### Assess non-specific effects through multiple methods and on an ongoing basis

Non-specific factors (such as empathy and components that lend themselves to the target communication style) will be assessed as a stand-alone domain within the RETURN checklist. Nonspecific effects will also specifically be discussed during shadowing training.

#### Ensure both adherence to the protocol and competency of intervention delivery

Adherence and competency of intervention deliveries will be assessed through the application of the RETURN checklist throughout the main trial. In addition, 100% of all available recordings will be assessed at the end of the main trial to provide a comprehensive overview of the adherence and competency of intervention deliveries. A fidelity threshold of 80% in every domain per intervention delivery will be applied when scoring the recordings.

### Receipt

#### Ensure participants’ understanding of the intervention

Although data collected from the patients during the feasibility study suggested that patients overwhelmingly found the intervention useful, understandable and relevant, it is helpful to outline here the steps taken to enhance participants’ understanding of the intervention during its development:

- The RETURN intervention is designed to be engaging, specifically targeted to the trial population. An extensive patient and public involvement (PPI) work stream fed into its design (full details have been published elsewhere [18]), with the aim of ensuring the materials were culturally relevant, containing congruent messages and images to the trial population. A design company and a professional illustrator were employed to embed these strategies.
- To account for different learning styles, information was presented and repeated using multiple formats - verbal, written, pictorial and videography.
- The intervention materials were written to a reading age of 8 years to ensure health literacy inclusivity.
- Intervention delivery sessions are formatted as reciprocal conversations, and therefore by design, mutual understanding between the patient and dental nurse is embedded. During training and throughout the recruitment period, intervention deliveries will be scored, and feedback provided to ensure that ‘reciprocity’ and patient understanding is embedded, with these criteria factored into the RETURN checklist.

#### Ensure participants’ ability to perform behavioural skills

The RETURN intervention seeks to target the behaviour of routine dental appointment visiting. To ensure patients’ ability to perform the behavioural skills required, the intervention was designed to be tailored, considering obstacles unique to individuals’ lives. The intervention culminates in a goal setting and action planning exercise, where participants think through their individual circumstances, and write out SMART (specific, measurable, achievable, relevant and time-bound) [32] goals and plans to help them to overcome their barriers. In this way the target behavioural skills were articulated, discussed and broken down into small actions.

From the feasibility observations, this element of the intervention needed improvement, specifically around patient engagement.

The nurse put the booklet to one side, and then took the planning booklet from their knee. “We know that writing plans helps”. I felt this introduction didn’t really explain to the participant what the nurse was asking them to do – The nurse looked at me to help as they were getting their words muddled…The nurse devised the plan **for** the participant, rather than letting the participant make the plan for themselves. The patient set their goal themselves, but they did not put in much detail. They wrote down 3 words and didn’t discuss this with the nurse at all.

DN02, Observation 11

For the RETURN main trial, several strategies will therefore be implemented to improve how assessment of behavioural skills were conducted during the intervention delivery sessions:

- Training will include a dedicated component on how to facilitate goal setting and action planning, emphasising the importance of facilitating and not leading the task, and how to encourage patients to think through and articulate their own mechanisms.
- Goal setting and action planning have been included on the RETURN checklist, and timely feedback will be provided.
- A follow-up text message will be sent to participants a week post-intervention including the participants’ own wording from their goals and plans set within the intervention sessions to reinforce behavioural skills and build self-efficacy.
- The 6-month follow-up telephone call to patients will explore their comprehension of the intervention and how meaningful they found it to track receipt.
- A component of the intervention conversation will encourage discussion around what was achieved during the intervention session. This has been designed to improve participant receipt of the intervention by setting intentions. This element is also included in the RETURN checklist assessment.

### Enactment

#### Participant performance of the intervention skills will be assessed in settings in which the intervention might be applied

Data was collected from patients of the feasibility study amid the first COVID lockdown restrictions (May – September 2020), and accordingly it was not possible to assess enactment at that time. Therefore, as part of the RETURN main trial telephone follow-up at three time points, questions will be included about whether and how the intervention materials and associated intervention skills had been used since leaving the urgent care dental setting. Questions will focus on which parts of the intervention had been used, whether the intervention skills had been enacted (i.e. phoning for a dental appointment, exploring which dental practice they may like to contact, attending a dental appointment) and how the intervention supported any actions taken to attend a routine dental appointment.

Additionally, enactment strategies are embedded within the intervention materials themselves. Some materials are labelled ‘to look at at home’, providing encouragement and support in locating a dentist, making an appointment and thereafter attending an appointment – the behaviours targeted by the intervention.

The full RETURN fidelity strategy is summarised in Table 4. The strategies presented there show the tangible actions taken to attend to the various intervention fidelity recommendations, which may help other researchers to think through the strategies that will apply to their studies (i.e. using audio recordings to monitor skills drift).

**Table 4:** RETURN Fidelity Strategy Summary

BCC Recommendation	RETURN Strategy	Method (where applicable)
**Design**		
Explicitly identify and use a theoretical model as a basis for the intervention and ensure the intervention components and measures are reflective of underlying theory	State theoretical underpinnings of the intervention	Published paper outlining intervention development with logic model (18)
Ensure consistent intervention dose and develop a monitoring plan to maintain consistency	Specify target dose (15 min), provide timings breakdown of intervention components	Incorporate dose awareness into training. Dose is an allowable adaptation for RETURN, but dose will be explored in results
Develop a plan for how adherence to the protocol will be monitored. Monitor both intervention delivery and assessment administration	Recordings of intervention delivery sessions of each interventionist were assessed throughout the trial. 80% fidelity threshold within each intervention component required. A fidelity scoring guidance was developed	Audio-recordings of intervention delivery sessions. Iterative development of the scoring guidance with interrater reliability measures to ensure consistent assessment administration
Develop a plan to record intervention protocol deviations and a method for providing timely feedback to interventionists	Use a coaching style with training. Provide monthly feedback on sessions in a transparent way, using the RETURN checklist, supplemented with booster training and site support visits	Audio-recordings of intervention delivery sessions
Develop a user-friendly scripted intervention manual to ensure consistency of delivery and adherence to active ingredients of the treatment	Instead of scripts, the use of easy to digest crib sheets and supporting training materials designed to be used in intervention sessions	
Plan for implementation setbacks	Hire float dental nurses to carry out research tasks as needed, as well as to support nurses at sites. In addition, a plan to train multiple nurses at each site	
Minimize contamination between conditions	Training and the development of crib sheets that set out research activities for each arm. Questions were included in the trial follow-up to explore contamination with participants	Incorporate contamination awareness into training plans, including user friendly cribs sheets. Participant self-report at follow-up
**Training**		
‘Hiring’ dental nurses to deliver the RETURN intervention	Training to incorporate ‘soft skills’ necessary to foster intervention delivery skills and increase confidence. The use of a coaching style throughout the length of the trial	Observations
Standardise training	Use of ‘train the trainers’ model with the same team of trainers throughout the trial, use of identical training materials for all sites, the development of a central website to house all training materials, in addition to the provision of hard copies for all dental nurses	
Ensure dental nurse skill acquisition	The use of tests and pass scores for all training modules, utilising different methods (written, oral and action based)	
Minimise ‘drift’ in dental nurse skills	Assessment of intervention sessions for all nurses throughout the trial, triggered booster training, triggered or requested reflective practice sessions, coaching style maintained	Audio-recordings of intervention delivery sessions over time
Accommodate dental nurse differences	On-the-job shadowing training element, which provides real-time feedback and offers tailored training to suit individual needs	Observations
Enhance buy-in from dental nurses	CPD hours, training to include information about potential patient benefit, inclusion of soft skills development in dental nurse training, including dental nurses in early discussions with dental sites, regular engaging newsletters aimed at dental nurses, engagement lunches, regular site visits, use of communication modes congruent with dental nurse preferences, awards evening to reward achievements	
**Delivery**		
Use a scripted curriculum or treatment manual	Instead of scripts, provision of a training manual with cheat sheets, cribs sheets and videos. In addition, some of the intervention materials were used as a ‘script’ to enhance standardisation	
Assess nonspecific effects through multiple methods and on an ongoing basis	Scored using the RETURN checklist throughout the trial, with feedback provided where necessary	Observations and audio-recordings
Ensure both adherence to the protocol and competency of intervention delivery	Scored using the RETURN checklist, with 100% scored at the end of the trial to provide an overview of adherence and competency in the trial	Observations and audio-recordings
**Receipt**		
Ensure participants’ understanding of the intervention	The intervention was developed with the target population at the heart of its design, ensuring the intervention was engaging, culturally relevant and aesthetically pleasing. The intervention was presented in multiple formats to engage different learning styles. The intervention materials were developed to an appropriate reading age to facilitate health literacy inclusivity. Reciprocity is embedded within the intervention delivery, and enhancement of patient understanding embedded within the structure of the intervention session	Observations, audio-recordings
Ensure participants’ ability to perform behavioural skills	Goal and action plan facilitation is a component of the intervention where behavioural skills will be articulated. Training focuses on how to facilitate tailored goals and action plan setting. A follow-up text message forms part of the intervention to encourage behavioural skills. Questions around behaviour skills forms part of the trial follow-up with participants. Intention setting is included as part of the intervention where behavioural skills will be reaffirmed. Audio-recordings were scored against the RETURN checklist	Observations, audio-recordings, participant self-report
**Enactment**		
Participant performance of the intervention skills will be assessed in settings in which the intervention might be applied	Questions were included in the 6-month telephone follow-up around enactment of the intervention skills	Participant self-report

## Discussion

This article presents a comprehensive fidelity strategy to be embedded within the RETURN main trial. To the best of the authors’ knowledge, this is the first published fidelity strategy for the testing of a BCI in the primary care dental setting. This strategy has sought to balance the needs of both the research and the dental practice context.

Research has shown that outcomes are improved when interventions are delivered with a high degree of fidelity [33–36], and one review found that effect sizes are at least 2 to 3 times higher when interventions are delivered with high intervention fidelity [29]. In addition, by devising and implementing a robust fidelity plan, theoretically this allows for the assessment of ‘infidelity’ and for exploration of how differences in fidelity may be associated with outcomes [25]. The development of a comprehensive fidelity strategy for use in the RETURN main trial therefore seeks to provide the methodological assurances necessary to determine whether the RETURN intervention is effective or not.

In addition, published fidelity strategies can serve as blueprints for other researchers, enabling the replication of interventions across various settings or populations [37]. This facilitates accurate implementation and consistency among studies, ultimately promoting the reproducibility of findings. Furthermore, the dissemination of fidelity strategies enhances transparency and accountability in research, allowing stakeholders—including funding agencies, peer reviewers, and the broader scientific community—to assess the rigor and validity of study methodologies, thereby ensuring ethical conduct and integrity in research practices [38]. We would encourage the publication of fidelity strategies as a way of sharing best practice to others in the field.

One strong message from the findings of the feasibility analysis was the importance of the role of the research team in dental research. It is clear from this study that research teams facilitating BCIs in the primary dental care context need to be mindful of the constraints of the setting and the pressures and skill mix of the healthcare professionals within it. Whilst the primary aim of the strategies developed for the RETURN main trial is to enhance intervention fidelity, a secondary aim of the selected strategies is to minimise burden for the dental teams involved. This is important as the primary dental care setting routinely incurs challenges such as time and staffing limitations [14], and for sites working to provide urgent clinical care in a target driven remuneration system (such as the NHS) as in RETURN, there are additional pressures [39] which may well have been exacerbated post-COVID-19 [40]. An example of this ‘shift’ in burden is where the decision made to audio-record all of the intervention delivery sessions to monitor skills and assess delivery fidelity (rather than other methods such as asking the dental nurses to complete check-lists as has been adopted in other primary care dental trials [41]). This will reduce the time and process burden on the dental nurses within the wider context of the RCT which, outside of the intervention delivery, has its own lengthy procedural requirements (e.g. consenting, randomising, data collection, data entry etc.). Additionally, the use of audio-recordings for fidelity monitoring is considered the gold-standard [1], and whilst acknowledging that this method is researcher resource intensive, it is deemed the most appropriate method for use in the primary dental care context.

This publication provides a thorough description of the RETURN fidelity strategy, which should be considered alongside the RETURN main trial results when they are published to assure the scientific integrity of our research practices.

### Limitations

This study has several limitations. Observations and interviews were conducted at only two sites with two dental nurses due to the small-scale nature of the feasibility study and early termination due to the COVID-19 pandemic. This means that a narrow range of perspectives were included in the findings used to develop the fidelity strategy.

Additionally, several BCC recommendations [1, 8] were not fully implemented in the RETURN fidelity strategy. Specifically:

1. **Monitoring of Control Participants**: The fidelity plan did not include monitoring control participant activities, although post-delivery participant self-report contamination assessments were conducted. This decision was made to enhance the acceptability of audio recordings among dental teams and patients. Only intervention delivery sessions were recorded, with control group conduct comprehensively covered in training.
2. **Protocol Review Group**: A protocol review group was not established to ensure the active ingredients of the intervention were fully operationalised due to resources limitations. Nevertheless, the intervention and training plan received input from two psychologists who were part of the RETURN team.
3. **Matching Interventionist Characteristics**: Due to constraints within the setting, it was not feasible to match key characteristics of the trial population with those delivering the intervention. However, a deliberate choice was made to involve dental nurses rather than dentists in the study design to facilitate rapport building. This decision was support by PPI work.
4. **Use of Independent Coders**: The use of independent coders was not feasible for the RETURN main trial due to resource limitations. However, as per recommendations by Borelli [8], the coding team was blind to outcome data.
5. **Pre and post-test measures**: To minimise dental staff burden and maintain proportionate measures, pre- and post-test process and knowledge assessments were not utilised in the RETURN main trial.

The RETURN feasibility study was conducted in England, UK within the context of the NHS primary dental care system. Within this system, the dental sites involved in the delivery of the RETURN research were providing commissioned urgent dental care in accordance with agreed General Dental Services (GDS) contracts. GDS contracts require a pre-defined number of Units of Dental Activity (UDAs) to be fulfilled within a year and for an agreed remuneration value [42]. However, any unmet clinical delivery targets can result in financial claw-back. Given that within this system, there was no additional capacity allocated for the delivery of the RETURN research, there was potential for tension between clinical contractual obligations and research delivery. Consequently, the RETURN fidelity strategy was developed with this context in mind.

Whilst the dental practice owners were financially compensated for staff time spent on the research delivery, no additional levers were in place to build research capacity, with those ‘doing’ the research often delivering their usual role alongside. This resulted in challenges and meant that the RETURN research team needed to be mindful of the context, and the fidelity strategies developed. In addition, those delivering the research (in this case dental nurses) were not directly in receipt of any remuneration for their additional efforts, and this was also considered during the development of our strategy.

Comparisons of this context to other primary care dental health systems across the globe suggests alternative fidelity strategies could be employed to best suit different contexts, and that the fidelity approach laid out within the paper may need to be adapted to better suit each context. For example, in some States in the United States of America (USA), the dental health system is solely privately funded [43]. A private dental health system, not constrained by targets in the same way as the GDS contracts within the NHS, in theory, could have more scope for research taking place alongside clinical delivery without it being squeezed out by business pressures. This could lead to a lighter-touch fidelity strategy employment. In addition, schemes such as the National Dental Practice-Based Research Network in the USA or the Australian Dental Practice-Based Research Network which build capacity for research delivery within dental practices could play a key role in research facilitation which again, may alter fidelity strategy approaches.

Finally, an important consideration to acknowledge is what it means for an RCT where researchers intervene to influence intervention fidelity. It could be argued that by manipulating intervention fidelity it changes the implementation landscape to an extent that recorded outcomes are no longer the simple effect of trial allocation, and therefore, that this may not be representative of outcomes that would be achieved in the real-world [25]. However, for reasons described throughout this article, the primary dental care environment is unique and challenging, and therefore the decision was taken to ensure that intervention fidelity will be closely monitored and improved throughout the RETURN main trial. Dental teams can be inexperienced with conducting research trials [44], and researchers also have a responsibility to those involved in the conduct of research to provide support and guidance to ensure the best outcomes for the trial.

### Conclusions and implications

The fidelity strategy outlined in this paper serves as a blueprint for researchers conducting BCI trials in primary dental care settings. This environment presents unique challenges, necessitating a contextually informed approach to enhance fidelity. However, many strategies detailed herein could be transferable to other BCI trials in similar contexts, despite being specifically tailored for the RETURN trial. The RETURN fidelity strategy summary could be a useful tool for other BCI trialists in the primary dental care setting, as this provides tangible examples of how the strategies were operationalised. We image that the extent of these strategies will alter depending on the context, resource availability and the intervention itself. This publication outlines a best practice approach and should be read in conjunction with the forthcoming results of the RETURN main trial.

## Electronic supplementary material

Below is the link to the electronic supplementary material.

Supplementary Material 1

Supplementary Material 2

Supplementary Material 3

Supplementary Material 4

Supplementary Material 5

Supplementary Material 6

## Data Availability

The datasets analysed during the current study are not publicly available as they are qualitative datasets that are bound by confidentiality and anonymity restrictions. Fully anonomised data, however, could be made available from the corresponding author upon reasonable request.

## Abbreviations

BCC: The National Institutes of Health Behaviour Change Consortium
RETURN: InteRventions to rEduce inequaliTies in the Uptake of Routine deNtal care
BCI: Behaviour change intervention
RCT: Randomised Controlled Trial
MRC: Medical Research Council
UK: United Kingdom
NHS: National Health Service
MI: Motivational Interviewing
SD: Standard Deviation
SMART: Specific, measurable, achievable, relatable, time-bound
PI: Principal Investigator
CPD: Continuing Professional Development
PPI: Patient and public involvement
GDS: General Dental Services
UDAs: Units of Dental Activity
USA: United States of America
