# Supplementary material for: Developing intervention fidelity strategies for a behaviour change intervention delivered in primary care dental practices: the RETURN fidelity strategy
Source: BMC Prim Care. 2025 Feb 17;26:43. doi: 10.1186/s12875-025-02732-1 (PMC11831780; doi:10.1186/s12875-025-02732-1)
Supplement: Supplementary file 1 — Supplementary Material 1 [file 12875_2025_2732_MOESM1_ESM.pdf]

RETURN Scoring Guidance Manual

Criteria Parameters

Leniency should be applied with reviewing sessions with patients who do not engage with the intervention delivery or the delivery nurse. Nurses should be seen / heard to attempt the required 5 steps with all patients, plus the overarching principles, but the patient response should be taken into account with marking. i.e. open questions should be attempted, but if closed questions are resorted to, this wouldn't be a mark down if the patient is unresponsive. We are marking the nurse, not the patient.

Overarching Domains

- **Use of empathic listening statements:** empathic listening statements go beyond merely just hearing words, and the nurse should demonstrate that they actively understand the patient's emotions and perspectives. It should involve active listening which means the nurse should be full present, paying attention, and showing genuine interest in what the patient is saying, and empathy should be demonstrated by the nurse in their communication style by showing care and concern.

  We are looking for nurses using empathy in their conversations, reflecting on what the patient is saying, and using statements that provide the patient with a compassionate (or caring) environment to share their stories.

  - Score 0 if there is no evidence of empathic statements
  - Score 1 if there is some demonstration, but where there are opportunities missed by the nurse throughout the intervention session
  - Score 2 if there are empathic listening statements embedded but where one or two opportunities were missed by the nurse to increase this
  - Score 3 if empathic listening statements were fully embedded during the intervention delivery.

- **Use relevant open questions:** as a rule of thumb, open questions cannot be answered with a yes or no response. These questions should be used appropriately to afford the patient the opportunity to speak freely.

  Open questions should be used by the nurses to encourage the patient to think about their barriers / open the discussion, and also throughout including during the goal setting if possible. Open questions should always be attempted in the first instance by the nurses, but if a patient is unresponsive, closed questions may be used without impacting on the score to fulfil the steps of the intervention delivery.

  - Score 0 if no open questions were used
  - Score 1 if few open questions were used
  - Score 2 if open questions were used, but where there were one or two missed opportunities to embed more
  - Score 3 if open questions were used fully throughout the intervention delivery (where appropriate to do so)
    Patient responsiveness should be considered when scoring here.

- **Use of non-judgemental language:** is an active process where a conscious effort is made to keep any criticism or accusation out of the discourse with the patient.

  Nurses need to demonstrate non-judgemental language throughout the session with the patient. Scoring here needs to reflect the importance of this criteria.

  - Score 0 where only judgmental language is used
  - Score 1 if just one or two non-judgemental statements were used, but that some language could be construed as judgemental

  - Score 2 if manly non-judgemental language was used, but where there were one or two missed opportunities for non-judgemental language use
  - Score 3 if non-judgemental language was use throughout the course of the intervention delivery

- **Use of non-directive talk**: patients should be encouraged to talk freely during delivery sessions. Too much interpretation or explanation by the nurse should be avoided, especially during the initial chat where the emphasis is on the patient determining the direction of the session. After the initial chat, non-directive principles should be applied when going through the rest of the session – patients should be allowed to talk about the materials freely during the entire session should they wish to do so. Flexibility should be applied depending on the extent the patient is engaged with the session.

  This criterion can depend on patient responsiveness, and some leniency can be applied as some patients will need more guidance than others. This scoring should be based on appropriateness and opportunity afforded to the patient to direct the course of the session.

  - Score 0 if no attempt made to be non-directive i.e. the nurse speaks over the patient and directed the whole session without patient involvement
  - Score 1 if some attempt made to be non-directive, but at times during the session, the nurse directed the patient
  - Score 2 if there were one or two occasions when the nurse was directive, but overall was a non-directive session
  - Score 3 if non-directive talk was fully embedded throughout the whole session

- **Patient's priorities, beliefs and challenges acknowledged**: patients should not be challenged on their beliefs, priorities or challenges experienced previously, even if they are in direct conflict with the principles of the delivery nurse. These should simply be acknowledged as an experience that occurred.

  - Score 0 if patient's priorities/beliefs are challenged by the nurse e.g. Patient: "I couldn't get a dentist because there weren't any" Nurse "There was loads of NHS availability a year ago so that can't be true".
  - Score 1 if some attempt is made to acknowledge but the patient's priorities/beliefs are also challenged e.g. Patient "I couldn't get a dentist because there weren't any" Nurse "It sounds like it was really difficult for you to get yourself into the dentist, but there were dentists available".
  - Score 2 is patient's priorities/beliefs and challenges are acknowledged during most of the session, but once or twice the nurse challenged the patients on these.
  - Score 3 if acknowledgments rather than challenges are present. Patient: "I couldn't get a dentist" Nurse: "sounds like it was really tricky for you to get into a dentist in the past".

Step 1: Discuss Barrier – Raise Awareness

- **Patient given opportunity to tell their story (if they choose to do so):** the patient is in control of how much or how little they discuss in the session. However, each patient should be given the **opportunity** to tell their story, uninterrupted, and to receive encouragement to do so by the nurse.

  - Score 0 if the patient is not given any opportunity to tell their story during the delivery session
  - Score 1 if the patient is given some space to tell their story (and some encouragement to do so), but the nurse ends the conversation where more opportunity for discussion could have been afforded to the patient
  - Score 2 if the nurse cuts the patient short on one occasion during the session
  - Score 3 if the patient is given lots of opportunity to discuss their barriers, and how they feel about going to the dentist throughout the delivery

If a patient chooses not to tell their story, this should not impact this score if it is judged that opportunity for discussion has been afforded. When scoring, listen to what the patient is saying – make a judgment on whether the patient could have been encouraged to engage more, or whether the patient was not willing to engage any more.

- **Patient encouraged to come up with their own barrier(s) without being led or spoken for:** patients should lead delivery sessions as much as possible, with the nurse facilitating the conversation rather than leading it. Patients should be encouraged to come up with their own barriers first, without the nurse 'matching' them too soon. For example, if open questions have been tried, but the patient hasn't specifically stated one barrier that they wish to work on, it is appropriate to show the barrier booklets to the patients to ensure the matching is correct. Asking some probing questions is appropriate here to ensure the barrier has been decided upon by the patient, and not the nurse.

    - Score 0 if the nurse decides for the patient what their barrier is (without trying to engage the patient in the process at all)
    - Score 1 if the patient starts to come up with the barriers, but the conversation is taken over by the nurse to decide on a barrier
    - Score 2 if the patient discusses the barrier they wish to select, but the nurse is leading the conversation too much
    - Score 3 if the patient comes up with their own barrier, and the nurse's role is as facilitator only

    If a patient chooses not to engage in the process, this should not impact on the score if it is judged that encouragement was provided to the patient to come up with their own barriers -listen to the nurses behaviour.

- **Patient chose a barrier to discuss within the session and encouraged to decide on the one barrier they want to work on (if they have more than one):** due to time constraints, only one barrier should be selected to focus on in the session **by the patient**. If a patient has more than barrier, a discussion about which to focus on should be led by the nurse.

    - Score 0 if no discussion is held about focusing on one barrier to discuss in the session, or if no barrier is selected at all
    - Score 1 if the nurse decides which barrier to focus on (based on the previous discussion with the patient), rather than the patient
    - Score 2 if the patient selects the barriers that apply to them, but the nurse directs the patient to a barrier to focus on from the selections made by the patient
    - Score as 3 if the patient decides themselves on the one barrier they wish to focus on

- **Patient Pack given to patient:**

    - Score 0 if the pack is not given
    - Score 3 if the pack is given

Step 2: Increase Motivation - Video

- **Patient is shown the video relevant to their selected barrier:** patients should be shown the video that is relevant to their barrier ideally at the start of the session, immediately following the conversation about their barrier, as we know that patients remember information better that was given at the start and end (primacy and recency effects).

    - Score 0 if no video is shown.
    - Score 1 if a full video is shown that does not relate to the selected barrier
    - Score 2 if the relevant barrier video is shown to the patient, but not at the start of the session
    - Score 3 if the relevant barrier video is shown to the patient at the start of the session

- **Encouragement provided to the patient to reflect on the video, and how their own situation relates:** the point of this step is to increase the patient's motivation. This is done here by asking the patient to reflect on the selected intervention video shown during the session, within which someone talks about their experience of overcoming their barriers. We are looking for a statement along the lines of "What did you think about that video, and how does that relate to your own situation?". If the patient is engaged, follow-up questions can be added "What did you think about what they said about overcoming their barriers, does that seem like something you could try?"

    - Score 0 if no encouragement to reflect on video is present
    - Score 1 if encouragement to reflect is provided but patient not given the space to do so before the session is moved on
    - Score 2 if some encouragement is provided, but patient is engaged and more conversation around the video could have been forthcoming
    - Score 3 if patient is encouraged to reflect on the video about how this relates to their own situation

Step 3: Increase Knowledge – Barrier Booklets

- **Relevant barrier booklet selected by the nurse:** participants should be directed towards the booklet that is relevant to their selected barrier, following on from the conversation and video – the barrier should match.

    - Score 0 if no booklet is selected
    - Score 1 if the patient is directed to the 'incorrect' booklet
    - Score 2 if the participant is directed to their relevant booklet, but the nurse isn't clear about that when completing this step
    - Score 3 if the participant is directed to their relevant booklet

- **Relevant booklet content provided to the patient:** participants should be shown the booklet content that is <u>relevant</u> to them and their barrier. Participants do not need to be shown the whole booklet, but they should have the pertinent parts of the booklet explained to them. As a minimum, nurses should draw the participant's attention to one or two relevant parts of the booklet. The aim is to cover all relevant sessions in the booklet with the patients. The nurses' own experiences relating to the booklet content are also relevant here. The participants get the booklets to take home with them, but some effort should be made to read out one or two of the relevant pages (based on their previous conversations) to the patient.

    - Score 0 if no booklet content is discussed
    - Score 1 if some booklet content is discussed, but this was not done in detail, and the booklet messages themselves were not used at all i.e. if a page was discussed <u>only</u> from the nurse's own experiences
    - Score 2 if most of the relevant booklet content was covered by the nurse, but some other parts would have been relevant based on the prior conversations
    - Score 3 if the relevant booklet content is discussed, with all the pertinent pages / messages being read out to the participant, enhanced with nurse experiences

- **Statements communicated offering hope and assurances to the patients about their ability to overcome barriers:** this is about providing a statement or two reassuring the patient about their ability to attend future dental appointments and overcome their barriers. This could be done through using the nurse's own experiences, e.g. "You can agree on a stop signal like raising your left hand. I always check the patient's left hand then, and this can help you to feel more in control". Specifically, it's about making this relevant to the patient's barrier, and also specific issues they have with attending the dentist. E.g. "I really believe that you'll be able to overcome your anxieties, and this chat is the first step".

    - Score 0 if no hope or assurances provided by the nurse

- Score 1 if a statement offering hope and assurances is provided, but this is not tailored to the patient' circumstances
- Score 2 if a tailored hope and assurances statement is provided but where the number of statements could have been increased / delivered more authentically
- Score 3 if hope and assurances are offered to the patient specifically citing the patient's barrier(s) when providing this, and this is done in an authentic style

- **Emphasis placed on the benefits of regular dental attendance:** this relates to the nurse advising the patient about the benefits of regular dental attendance, and how regular attendance could benefit the patient, and is recommended. This can either be solely guided by the booklets, or by nurses adding in their own experience too.

  - Score 0 if there is no emphasis placed on the benefits of regular dental attendance
  - Score 1 if there is some emphasis placed, but it is judged that there could have been more
  - Score 2 if the benefits of regular dental attendance are communicated to the patient, but the messages are not tailored to the participant (e.g. for an anxious patient, one of the benefits of regular attendance is the building of rapport with a dental team, thus lowering anxiety etc.)
  - Score 3 if there if it is judged sufficient emphasis was placed on the benefits of dental attendance, tailored to the patient

Step 4: Setting a goal and action plans

- **Patient set a goal and action plan:**

  - Score 0 if no goal or action plan are set
  - Score 1 if only a goal **or** action plan set
  - Score 2 if a partial goal and action plan is set
  - Score 3 if both a goal and action plan are set

If a participant chooses not to engage with any part of the goal and action plan, or part of the action plan is not relevant to them (e.g. they do not have anyone in their social circle who could help them overcome their barrier) this should not impact on the score, this is about the nurse encouraging and supporting the patient to set their goal and action plan.

- **SMART principles applied to help the patient set a goal and make an action plan that is tailored to suit their situation:** The main principles here are about making a plan that is relevant to the patient's life (Specific, Measurable, Achievable, Related, Time-limited). Participants might find this challenging, but it is not the participant responsiveness we are scoring, it is the comments and support offered by the nurse to try to elicit a SMART goal from the participant / the way the task is explained by the nurse that is being scored.

  - Score 0 if no attempt is made to incorporate SMART principles
  - Score 1 if some attempt to use SMART principles are applied but only to the goal OR the action plan
  - Score 2 if some SMART principles are encouraged, but this could have been increased to facilitate the goal and action plan task
  - Score 3 if SMART principles are fully encouraged by the nurse (whether the patient engages with this or not)

- **Patient encouraged to set their own goal and make their own action plan:** The word **encourage** here is the key. We are looking for patients to be encouraged to set their own goals and action plans whilst being mindful that not all patients will want or be able to do this.

  - Score 0 if no encouragement is offered whatsoever to the participant to set their own goal and plan.

- Score 1 if it is judged that some attempt is made to encourage the patient to set their own goal or create their own action plan
- Score 2 if some encouragement is provided to the patient to set their own goal and action plan, but the encouragement could have been increased.
- Score 3 if the patient was fully encouraged to set their own goal and action plan.

Resorting to using the goal and action plan examples in the barrier booklets would not preclude a score of 3 if an attempt is made to encourage the patient's free thinking.

Step 5: Increase Intention

- **Encouragement statements communicated to the patient**: this is about the nurse providing encouragement to increase engagement with the intervention, and thereafter to increase the patient's intention to enact the skills learned during the delivery session. Encouraging statement can be about how well the patient has done in the session e.g. "I hope you manage to get yourself back into routine care. Remember to look back at your plan, as I think you've made a good first step". It could also be about future dental visiting e.g. "I really hope you manage to get yourself back into dental care and overcome your distrust of dentists, remember this was just the first step". It could also be about using the pack in the future to get themselves back into routine care. This step does not have to appear at the end, and this can occur throughout the session.

    - Score 0 if no encouragement statement is made
    - Score 1 if a small amount of encouragement is provided, but this could have been increased.
    - Score 2 if good encouragement statements are offered, but where these could have been tailored slightly more to the patient
    - Score 3 if the nurse provides an appropriate amount of encouragement throughout the session, tailored to suit the participant.

- **Encouragement provided to the patient to look at other intervention materials at home**. The nurse should direct the patient to the 'to look at at home' materials i.e. the next appointment toolkit, the employer card, the covid leaflet. This also could include the other barrier booklets and videos where a patient has more than one barrier and this seems relevant. Remember, the key thing here is relevance to the patient. All patients should have the next appointment toolkit, but the employer card might not necessarily be relevant for all (if the patient is not working).

    - Score 0 if no materials are pointed out to the patient
    - Score 1 if some materials are pointed out, and some materials that are irrelevant are discussed e.g. showing the employer card to a participant who has discussed they are not working.
    - Score 2 if most of the relevant materials were discussed, but one was missed out.
    - Score 3 if all relevant materials were discussed.
