# Supplementary material for: Developing intervention fidelity strategies for a behaviour change intervention delivered in primary care dental practices: the RETURN fidelity strategy
Source: BMC Prim Care. 2025 Feb 17;26:43. doi: 10.1186/s12875-025-02732-1 (PMC11831780; doi:10.1186/s12875-025-02732-1)
Supplement: Supplementary file 2 — Supplementary Material 2 [file 12875_2025_2732_MOESM2_ESM.pdf]

Intervention Delivery Steps to Follow:

1

Barrier Chat
- Get to know your patient
- Find out what’s stopped them from visiting the dentist in the past
- Make them feel comfortable, ask lots of open questions

“What’s stopped you from going to the dentist in the past”

2

Show Barrier Video
- Show the video on the tablet
- Ask the patient: *“What did you think about the video? Did you relate to it?”*

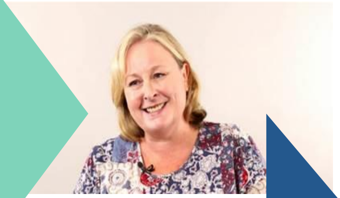

3

Read Barrier Booklet
- Select relevant information from the booklet to go through
- Use your own experiences as a dental nurse too

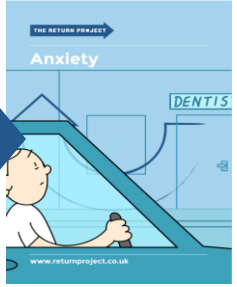

4

Make a Goal & Plan
- Work with the patient to write a goal and plan, using their words
- Make the goal and plan fit in with the patient’s life

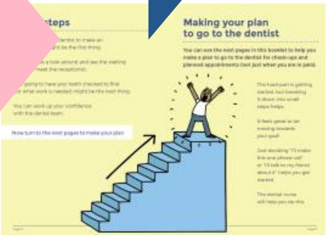

5

Provide Encouragement
- Let the patient know you believe they can reach their goals
- Tell the patient this has been a great first step for them

“You’ve done so well making your goal. I believe you’ll stick to it”
