# Supplementary material for: Developing intervention fidelity strategies for a behaviour change intervention delivered in primary care dental practices: the RETURN fidelity strategy
Source: BMC Prim Care. 2025 Feb 17;26:43. doi: 10.1186/s12875-025-02732-1 (PMC11831780; doi:10.1186/s12875-025-02732-1)
Supplement: Supplementary file 3 — Supplementary Material 3 [file 12875_2025_2732_MOESM3_ESM.pdf]

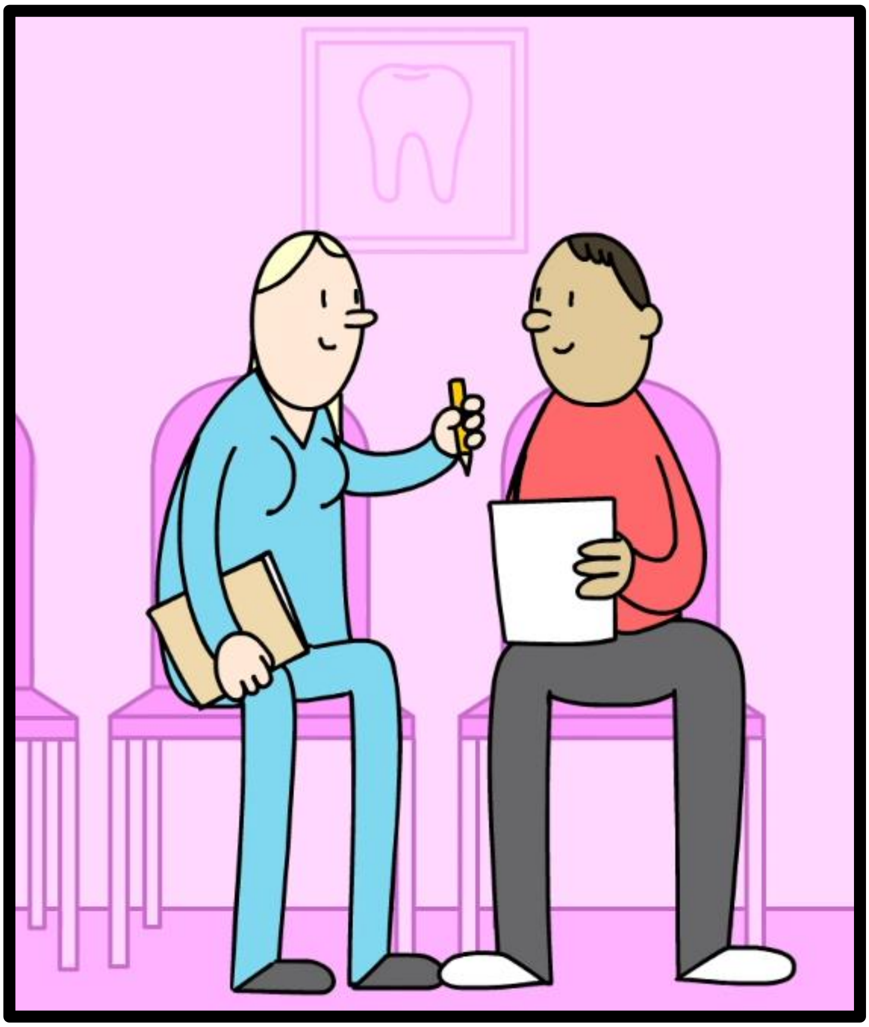

Intervention Facilitator’s Manual

Intervention Delivery Steps:

1.
- Discuss Barriers

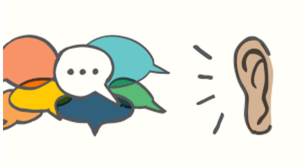

2.
- Increase Motivation

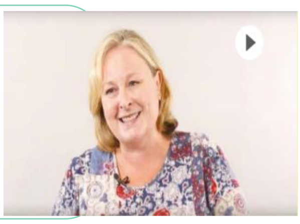

3.
- Increase Knowledge

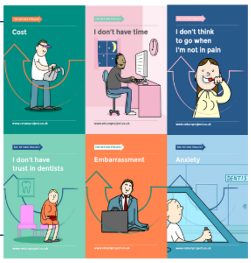

4.
- Set SMART Goals and Action Plan

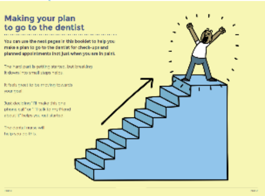

5.
- Set Intention

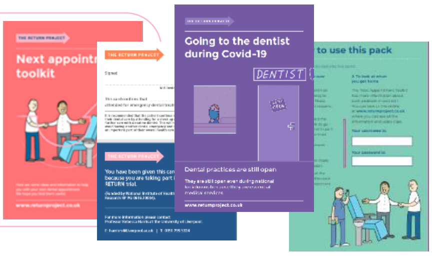

What is the aim of the RETURN Intervention?

The aim is to promote planned dental attendance by supporting adult patients to:

1. Identify reasons (barriers) they find it hard to attend the dentist for planned appointments

2. To help them work out ways to overcome their barriers.

This is done through a 15-minute personally tailored behaviour change conversation between a trained dental team member and a patient, using the intervention materials developed for this reason

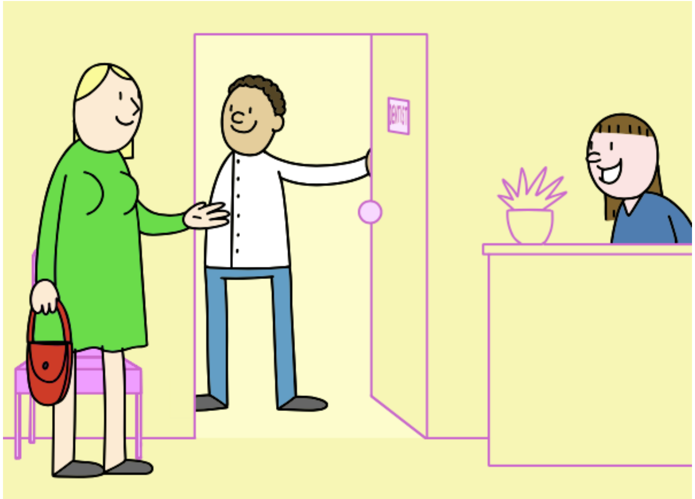

Step 1. Discuss barriers to regular dental attendance – raise awareness

What makes it difficult for you to attend the dentist?

Is that what you want to focus on today?

1.

4 mins

Discuss with the patient their barrier(s) to attending planned dental appointments.

- This is your opportunity to build a rapport with the patient
- Show you are interested in their story
- Demonstrate empathic listening skills
- Try not to 'correct' their experience by stating what they should or shouldn't have done / do in the future

Remember:

- ✓ Patients can discuss as much or as little as they want. They set the agenda for this conversation.
- ✓ Patients often have more than one barrier, and the barrier they lead the conversation with may not be the one you end up discussing. Explore this with the patient as much as possible

You can use the questions at the top of the page, or use this space to write any conversation starters you find useful as you go along:

____

____

____

____

Step 2.

Increase Motivation

- Can influence this when the patient feels:
  - Not judged
  - Collaborative
  - Hopeful

Here is where you will show a video

3 mins

2.

Next you will enhance motivation and confidence in the patient. You will do this using positive statements, and through the video.

Remember:

- It's important that the patient does not feel judged
- Take a collaborative approach to the discussion
- Voice hopeful statements to increase motivation

1. Suggested positive statement (tailored for the patient):

"It sounds like it's been really difficult for you in the past, but today we are going to focus on the first steps to helping you overcome your barrier, and get you visiting the dentist more regularly"

Now log in to the **Intervention Site** using the patient's login; select the relevant barrier.

2. Suggested video introduction:

" We have a video here of someone who has experienced a barrier similar to the one you have described. In it they discuss how they feel about going to the dentist now they are dealing with it "

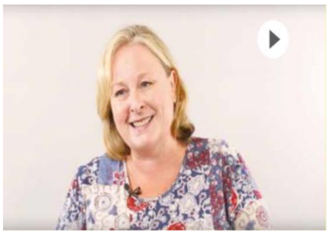

3. After the video, ask the patient what they thought about it.

Did the patient think it was useful? Did they relate to it?

Step 3.

Increase knowledge

- Provide logistical information based on one barrier
- Provide information on how to overcome barrier
- Offer hope and assurance of ability to remove barrier
- Emphasize benefits of regular dental attendance
- Provide social support

All of this is through conversation & barrier booklets

3 mins

3.

Use the Patient Packs to increase the patient's knowledge about the one barrier they have selected (there is lots of overlap however). The next pages of this manual describe what's in the packs and the individual booklets.

Pack Contents:

1. To look at during the conversation with patients

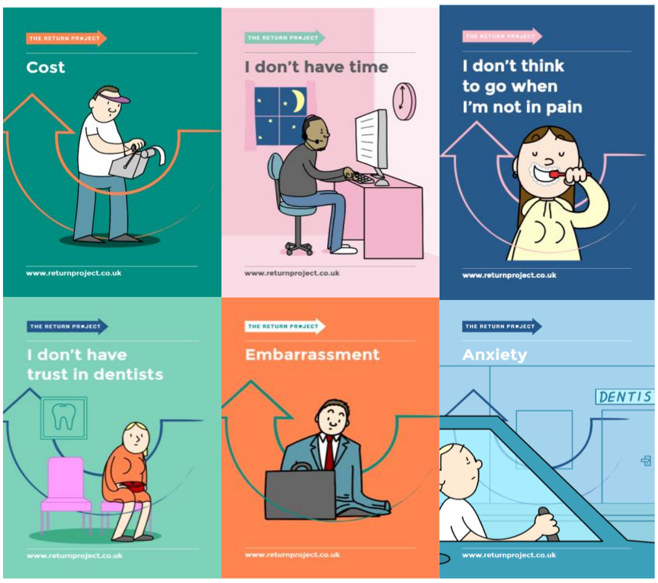

2. To be shown to patients, encouraging them to use these at home

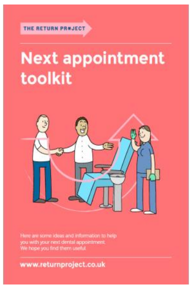

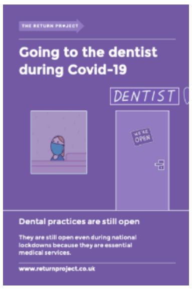

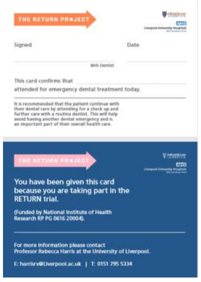

The Booklet

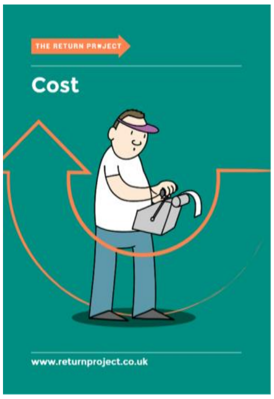

The Video

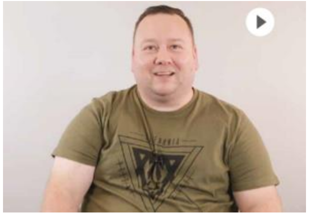

Additional materials you can direct the patient to

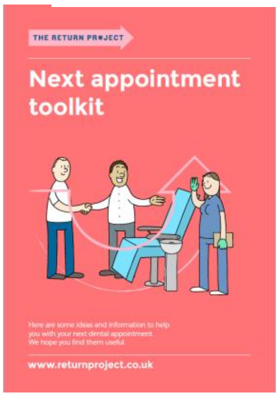

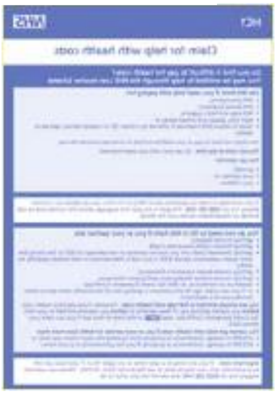

Booklet Page	Key messages about Cost
It doesn't cost as much as you think	• Watch Mike's video about cost<br>• Read Mike's quote on **p. 3** about thinking the costs would be really high
How much does it cost?	• Look at NHS bands and what each covers, **p. 4**<br>• People who need 5 fillings and 2 extractions pay the same (£65.20 in 2021) as people who need one filling. Even if they have lots of visits<br>• A check-up does not commit you to having anything done<br>• You can use the Next Appointment Toolkit to help you talk to the dentist about costs
Leaving things can cost you more	• Going to the dentist when you're not in pain can save you money in the long run
When money is tight	• Some people get help with costs of their dental treatment if they are on low income or benefits<br>• You do not pay anything if you are treated by dental students at the Dental Hospital<br>• The Next Appointment Toolkit will tell you how to get an HC1 form, and has more information about claiming costs

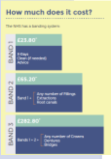

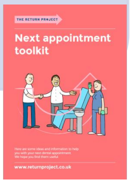

The Booklet	The Video	Additional materials you can direct the patient to
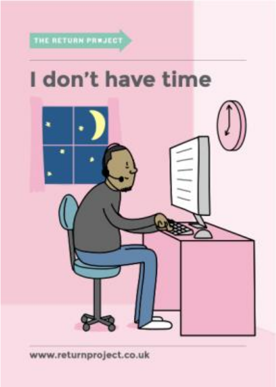	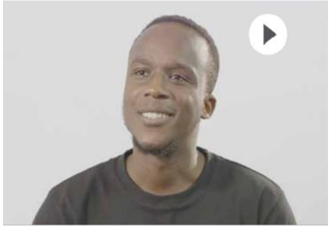	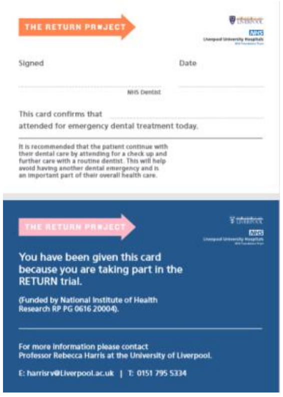 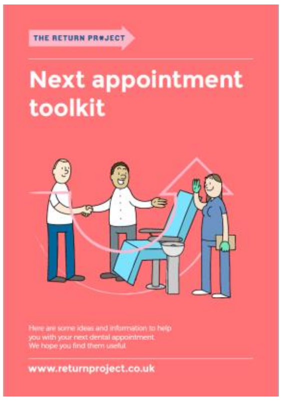

Booklet Page	Key messages about I don't have time
I can't take time off in the day	• If you find it hard to go to a dentist during the day, some NHS practices are open early morning and evening times. Some NHS practices even open on the weekend<br>• Extended hours NHS dental practices, **p. 4 & 5**
You can find a dentist which fits in with your life	• Getting a dentist is not like getting a doctor. You don't need to go to a dentist near your home if you don't want to.<br>• Your dentist can be near your work, or near a family member's house
Things happen	• Remember, you can always change your appointment if you can no longer keep it<br>• Don't give up. Make another appointment for a few weeks time when things are easier
My boss really doesn't like anyone being off	• Something that may help with asking for time off is to show your boss this card 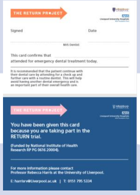
Planning a visit will help you find time when it's really difficult for you	• Some information in the Next Appointment Toolkit will also help you find a dentist and make an appointment 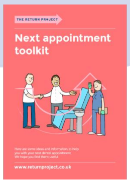

The Booklet

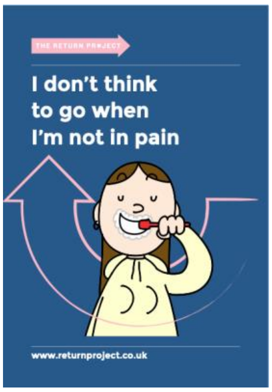

The Video

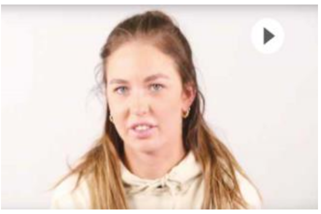

Additional materials you can direct the patient to

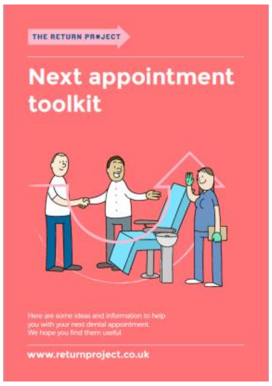

Booklet Page | **Key messages about I don't think to go when I'm not in pain**

For healthy teeth do I need to go?

Read Megan's story on **p.3**

Keeping on top of it

- People who don't see a dentist regularly are found to be 4 times more likely to have lost some of their teeth, than people who do
- Decay often starts between teeth where you wouldn't be able to see it yourself
- Look at the image on **p. 5**. I'ts too far gone to be helped by just toothbrushing

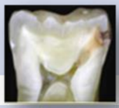

Mend it when you see it?

- Leaving it too long means you might end up needing more dental treatment or losing the tooth
- Having a full check-up with a dentist is the only way to see if you have other problems

Catch things early

- Mouth cancer is more common if people smoke and drink a lot
- Use the information in the Next Appointment Toolkit to help you make and keep your next appointment

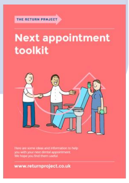

The Booklet	The Video	Additional materials you can direct the patient to
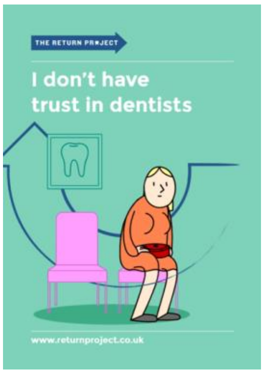	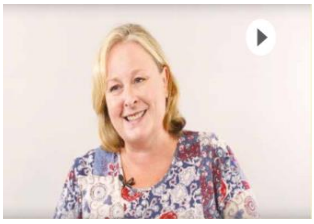	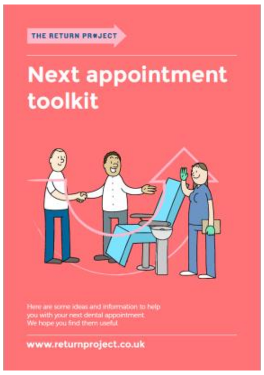

Booklet Page	Key messages about I don't have trust in dentists
Finding trust in a dentist	• Bad experiences at the dentist that people told us about were often many years ago. But treatment can be very different now. You can have a better experience next time<br>• Read Lisa's story on **p.3**
The dentist didn't listen to me	• Read Zach's story on **p. 4**<br>• Just because you had a bad experience with one dentist, doesn't mean that another dentist or visit would be the same
It's good to talk	• You don't have to have any treatment you don't want<br>• It's the dentist's job to make sure you understand what treatment you are having and why. It might be that you don't feel clear about things. On **p. 8** are some examples you could talk about with your dentist.<br>• If you are still not sure, you can ask for independent advice from the Dental Helpline at the Oral Health Foundation. Their contact details are on **p. 9**<br>• In the Next Appointment Toolkit there are some questions you can use: 1. at the reception about costs; 2. when you talk to the dentist at your check-up

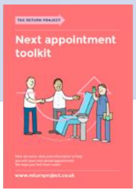

The Booklet	The Video	Additional materials you can direct the patient to
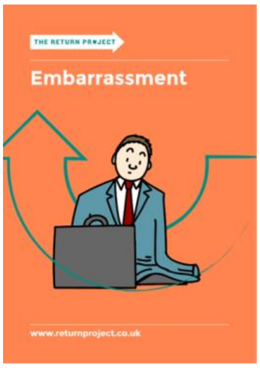	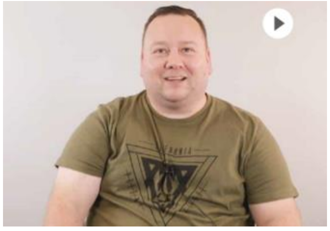	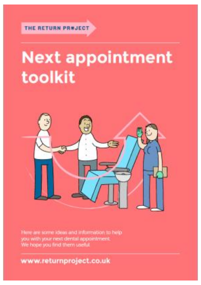

Booklet Page	Key messages about Embarrassment
I feel embarrassed that I've let my teeth go	• Quite a few people that we talked to felt embarrassed. You're not the only one!<br>• Read the story on **p. 2**<br>• Feeling regret about what has happened to your teeth is very common
I hate my teeth and worry what the dentist will think	• Dentists look at people's teeth all day long<br>• If you don't feel comfortable with a dentist, then you can always try another one
It makes me feel I'm a child	Read Mike's story on **p. 4**<br>• Just going for a check-up will remove the worry of not knowing!<br>• It might be difficult for you, but some of the hardest things in life are the most worthwhile<br>• Use the Next Appointment Toolkit to help you build confidence to go to the dentist 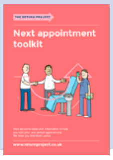
It's just history and you can move on	• Your teeth might have been damaged a long time ago. But that doesn't mean nothing can be done to improve your smile now<br>• You don't have to keep it to yourself. Visiting a dentist, even just for a check-up will help you know what can be done to improve your smile now
Getting sorted out can improve your confidence	• Read the story on **p. 7**

The Booklet	The Video	Additional materials you can direct the patient to
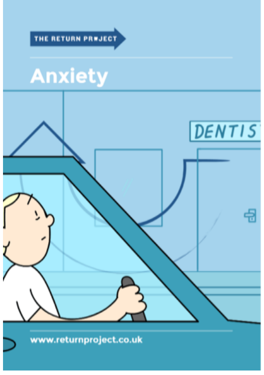	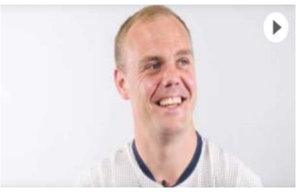	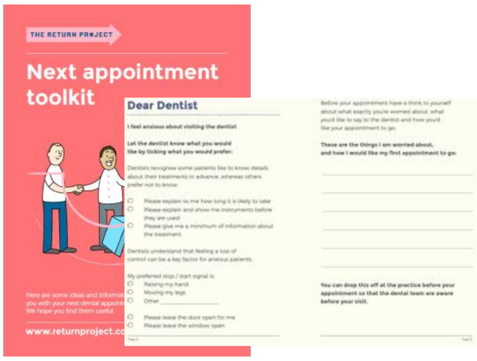

Booklet page	Key messages about Anxiety
Dental anxiety is common	• Up to 40% of people say they feel worried at the thought of having dental treatment. It's very common to feel like this so the dentist won't be surprised if you tell them that you feel worried about the visit.
Fears get built up over time	Lots of people go to the dentist and manage to have their teeth checked and fixed up
It's possible to get over dental anxiety	Just making that first visit and finding out that it's not that bad can make you much less worried
Telling the dentist helps<br>&<br>You are in control	• Talking to the dentist about what you are most worried about will help<br>• People often worry about not being able to stop the dentist when they are working on their teeth. But dentists are very used to this<br>• Remember you can ask your dentist to stop at any point
Dear Dentist	• Everyone is a bit different. On **p. 8 and 9** you can tick the boxes which you think would help you, then show it to the dental team. Let the dentist know what you would like<br>• You can also write down the things you are worried about, and how you would like your first appointment to go<br>• This is a page you can tear out and drop off at the practice before your appointment so that they are aware 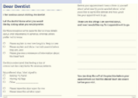
There are lots of things you can do to help	• Making your appointment for the first one of the day is a good idea.<br>• Look at the examples of things that can help you overcome your anxiety on **p.11**<br>• Use the Next Appointment Toolkit to help you overcome your anxiety 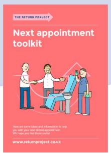

4.

Step 4. Setting SMART goals and action plans

Specific	• Goals & plans should be clearly defined
Measurable	• Goal & plan wording should allow patients to track progress – how will the patient know when their goal is achieved?
Achievable	• Goals & plans should be achievable for the patient
Relevant	• Goals & plans should be directly relevant to the patient
Time limited	• Goals & plans should set a definite deadline

4 mins

Next patients will make a goal and an action plan to overcome their barrier using SMART objectives. Turn to the end of the booklets.

1. Goal

Ask the patient to be specific in their goals: use events or time periods that are relevant to them, and achievable. They are more likely to achieve their goal if SMART objectives are followed.

> *"Thinking about how going to dentist would fit in with your life, when would be a good time for you to aim to make your next appointment by?– think about it in as much detail as possible, as you're more likely to achieve your goal if you do"*

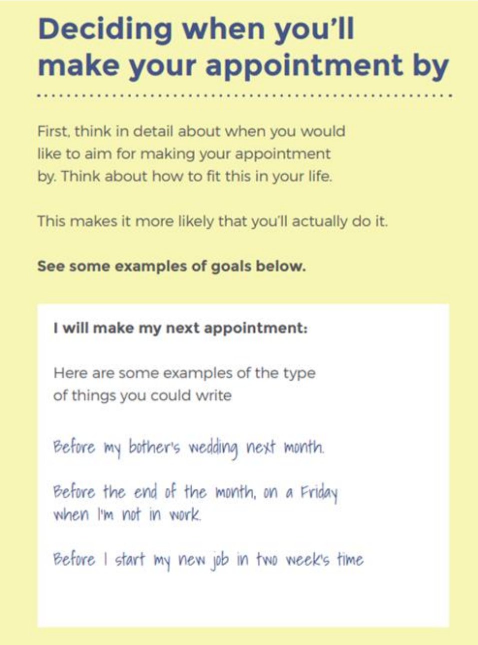

Deciding when you'll make your appointment by

First, think in detail about when you would like to aim for making your appointment by. Think about how to fit this in your life.

This makes it more likely that you'll actually do it.

See some examples of goals below.

I will make my next appointment:

Here are some examples of the type of things you could write

Before my bother's wedding next month.

Before the end of the month, on a Friday when I'm not in work.

Before I start my new job in two week's time

Looking at the examples here, you can see that these goals are:

- **Specific**: 'Before my brother's wedding'
- **Measurable**: 'Before I start my new job'
- **Achievable**: Goals are set by the patient, in a way that allows time that fits in with their own lives 'before I start my new job in two weeks' time'
  Relevant: Each goal should be relevant to the patient's life, and there should be detail of this 'on a Friday when I'm not in work'
- **Time Limited**: All goals should be set a time period 'Before the end of the month' or before a life event that's coming up that the patient will remember

2. Action Plan

Next is the action plan to help with the patient's identified barrier. Again, use the SMART objectives. The Action Plan has been split into three questions that ask patients to think about how to make their plan relevant to their life.

Each booklet contains an example plan for that barrier. You can read the examples out to the patient, if that would help.

Otherwise, work through each of the three questions to help the patient to set a SMART Action Plan.

Example plan for the barrier of anxiety

To help me to visit a dentist for a check-up and to attend planned appointments in the future, this is my plan.

To help me deal with the barrier of anxiety, I will:

Talk to other people about how I'm feeling. Let the dentist know I'm anxious when I go, and make sure we have a stop signal worked out at the start of the appointment.

"Now let's think about making a plan to tackle the barrier you've told me about today. Are there any techniques you've learned today that could help you, or is there anything else you want to write down here?
Again, think about what is most relevant to your life, with as much detail as possible as you'll be more likely to stick to it that way"

Thinking about what would work for me, the best time and place for me to do this is

When I'm feeling relaxed, which would be at the weekend. I can talk to my friend about it when we meet up for coffee next Saturday.

I will let the dentist know I am anxious when I telephone them, and I will also let them know at the start of the appointment.

"Next, think of where and when you feel it's best to do the things you've written down at the top.

Make sure you write down a time and place when you would feel comfortable doing this, so it's more likely you'll be able to achieve it."

Could somebody help you? Write about this here:

I will talk to the dental team about how anxious I am at the start of the appointment so that they can try to help me.

"Finally, is there somebody who could help you, or who you could talk to about this?

Try to be specific here, as it will help you to know who you could ask for help."

Remember to write the randomisation number on the goal and plan and take photos of both pages. Then upload these to REDcap.

Step 5. Intention

- Provide general encouragement to patient
- Provide encouragement to look at materials again when patient is home

Opportunity to make a difference!

Important that patient feels listened to, not judged and this is a collaborative process

1 min

5.

This final step in the intervention delivery is where you set the intention for the patient.

The essence of this step is to provide encouragement to the patient.

Remember:

- The last part of your conversation and the first part are what your patients will remember about the intervention session the most
- It’s important to leave them feeling positive about their encounter with you

Refer the patient to the other materials if you’ve not already done that in Step 3. Encourage them to look at the materials when they are home, and remind them they’ll get a text message to help keep their goal.

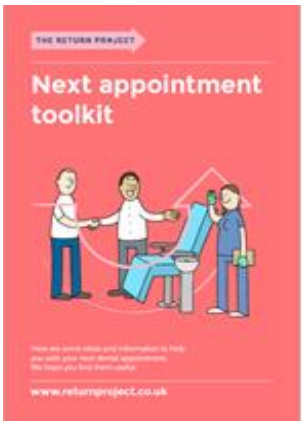

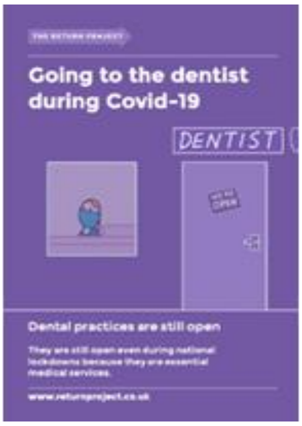

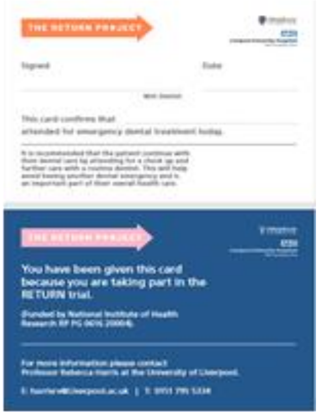

Suggested parting statement:

> *“The work we’ve done here today seems really important, and setting your goal and action plan is the first step for you. Well done. You’ve got these tools now to be able to go away from here and find a dentist that’s right for you.”*
