# Supplementary material for: Developing intervention fidelity strategies for a behaviour change intervention delivered in primary care dental practices: the RETURN fidelity strategy
Source: BMC Prim Care. 2025 Feb 17;26:43. doi: 10.1186/s12875-025-02732-1 (PMC11831780; doi:10.1186/s12875-025-02732-1)
Supplement: Supplementary file 4 — Supplementary Material 4 [file 12875_2025_2732_MOESM4_ESM.pdf]

Additional File 4

Training Content Checklist

**Training session date:**<br><br>**Trainers:**<br><br>**Site:**<br><br>**Attendees (name and job role):**		
**Training Component**	**Delivered**	**Not delivered**
Standardised RETURN intervention training slide desk used throughout the session		
A description of the RETURN intervention and the dental nurse role		
Overview of inequalities in dental attendance, and the health determinants that feed into this		
Group task designed to encourage thought around the steps and difficulties involved in behaviour change		
Overview of behaviour change theory with group discussion about personal experiences of this		
Overview of behaviour change conversations in a healthcare setting, with group participation		
Overview of effective communication skills with group discussions about personal experiences of this		
Interaction with the RETURN intervention materials		
Overview of SMART goals and action plans		
Role play exercise of intervention deliveries using at least 2 case vignettes		
Hard-copy training pack provided to all trainees		
Hard-copy patient intervention pack provided to all trainees		
Training website address details provided to all trainees		
