# Supplementary material for: Developing intervention fidelity strategies for a behaviour change intervention delivered in primary care dental practices: the RETURN fidelity strategy
Source: BMC Prim Care. 2025 Feb 17;26:43. doi: 10.1186/s12875-025-02732-1 (PMC11831780; doi:10.1186/s12875-025-02732-1)
Supplement: Supplementary file 5 — Supplementary Material 5 [file 12875_2025_2732_MOESM5_ESM.pdf]

Additional File 5

Intervention Delivery Training Assessment Checklist

Site Name:	**Score**			
**Training Component**	0	1	2	3
Demonstrated a basic understanding of factors that feed into health determinants				
Demonstrated a basic understanding of behaviour change conversations				
Demonstrated a basic understanding of how to convey emotional understanding of a patients' feelings and context				
Demonstrated a basic understanding of what good communication skills markers are				
Demonstrated a basic understanding of each of the RETURN intervention components				
Demonstrated intervention skills listed above through role play using case vignettes				
