# Supplementary material for: Developing intervention fidelity strategies for a behaviour change intervention delivered in primary care dental practices: the RETURN fidelity strategy
Source: BMC Prim Care. 2025 Feb 17;26:43. doi: 10.1186/s12875-025-02732-1 (PMC11831780; doi:10.1186/s12875-025-02732-1)

Small steps

Just going into the dentist to make an appointment might be the first thing.

You can have a look around and see the waiting area and meet the receptionist.

Just going to have your teeth checked to find out what work is needed might be the next thing.

You can work up your confidence with the dental team.

Now turn to the next pages to make your plan

Making your plan to go to the dentist

You can use the next pages in this booklet to help you make a plan to go to the dentist for check-ups and planned appointments (not just when you are in pain).

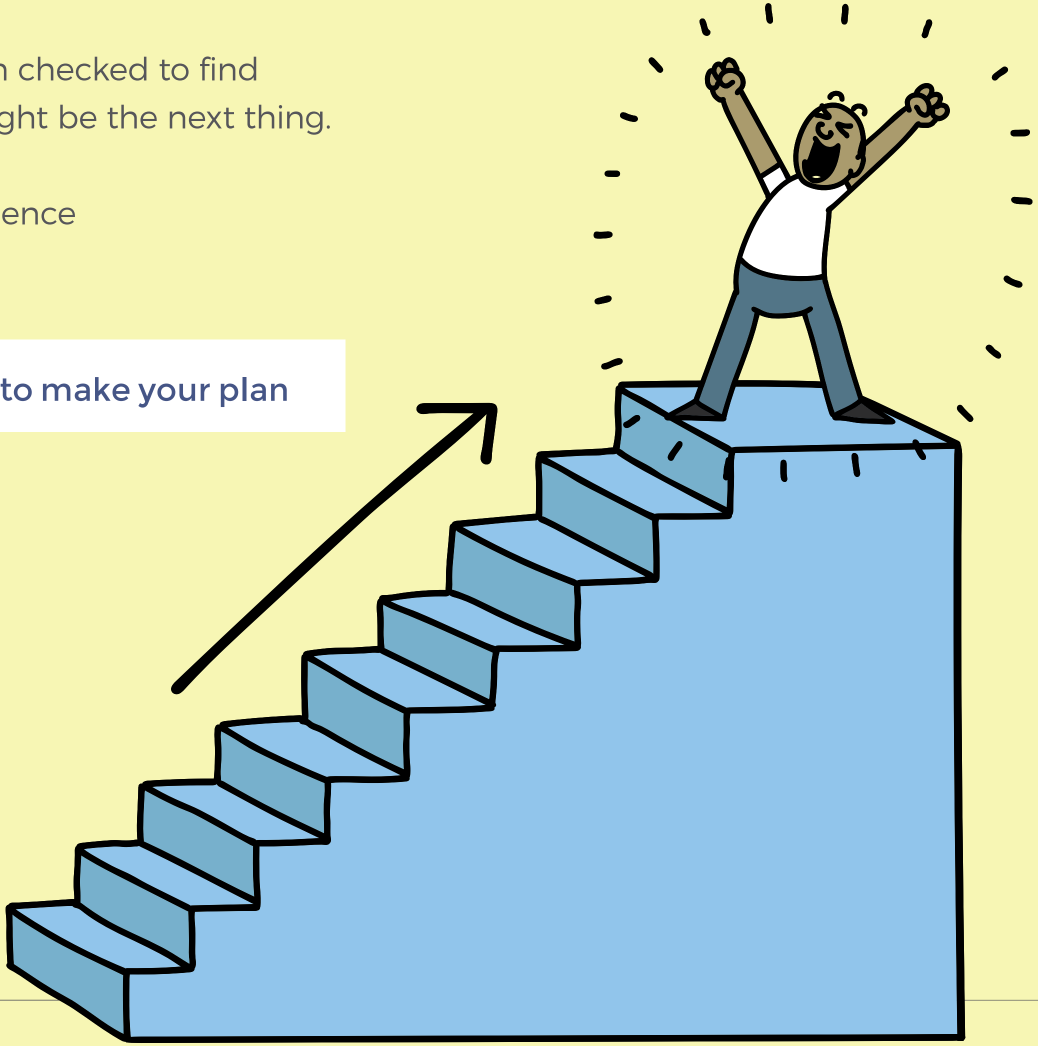

The hard part is getting started, but breaking it down into small steps helps.

It feels great to be moving towards your goal!

Just deciding "I'll make this one phone call" or "I'll talk to my friend about it" helps you get started.

The dental nurse will help you do this.

Deciding when you'll make your appointment by

First, think in detail about when you would like to aim for making your appointment by. Think about how to fit this in your life.

This makes it more likely that you'll actually do it.

See some examples of goals below.

I will make my next appointment:

Here are some examples of the type of things you could write

Before my bother's wedding next month.

Before the end of the month, on a Friday when I'm not in work.

Before I start my new job in two week's time

Setting your goal

Now write your goal

I will make my next appointment:

Randomisation Number

Example plan for the barrier of anxiety

To help me to visit a dentist for a check-up and to attend planned appointments in the future, this is my plan.

To help me deal with the barrier of anxiety, I will:

Talk to other people about how I'm feeling. Let the dentist know I'm anxious when I go, and make sure we have a stop signal worked out at the start of the appointment.

Thinking about what would work for me, the best time and place for me to do this is

When I'm feeling relaxed, which would be at the weekend. I can talk to my friend about it when we meet up for coffee next Saturday.

I will let the dentist know I am anxious when I telephone them, and I will also let them know at the start of the appointment.

Could somebody help you? Write about this here:

I will talk to the dental team about how anxious I am at the start of the appointment so that they can try to help me.

My plan for this barrier

To help me to visit a dentist for a check-up and to attend planned appointments in the future, this is my plan.

To help me deal with this barrier, I will:

Thinking about what would work for me, the best time and place for me to do this is:

Could somebody help you? Write about this here:

Well done for setting your goal and making your plan!

Remember, you can look back at this booklet at any time to remind yourself of your plan.

If you've given us your mobile number, we'll send you a text message soon to help you reach your goal of going to a regular dentist.

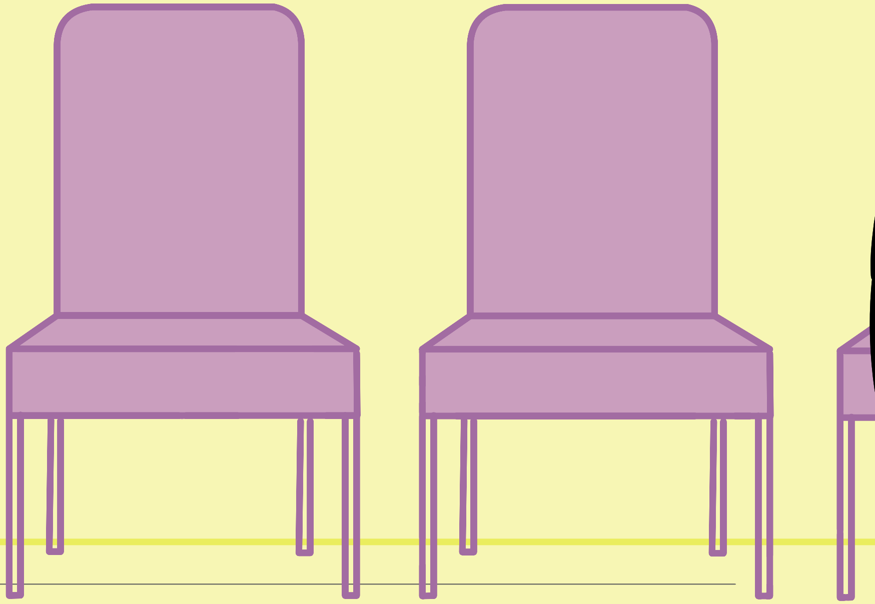

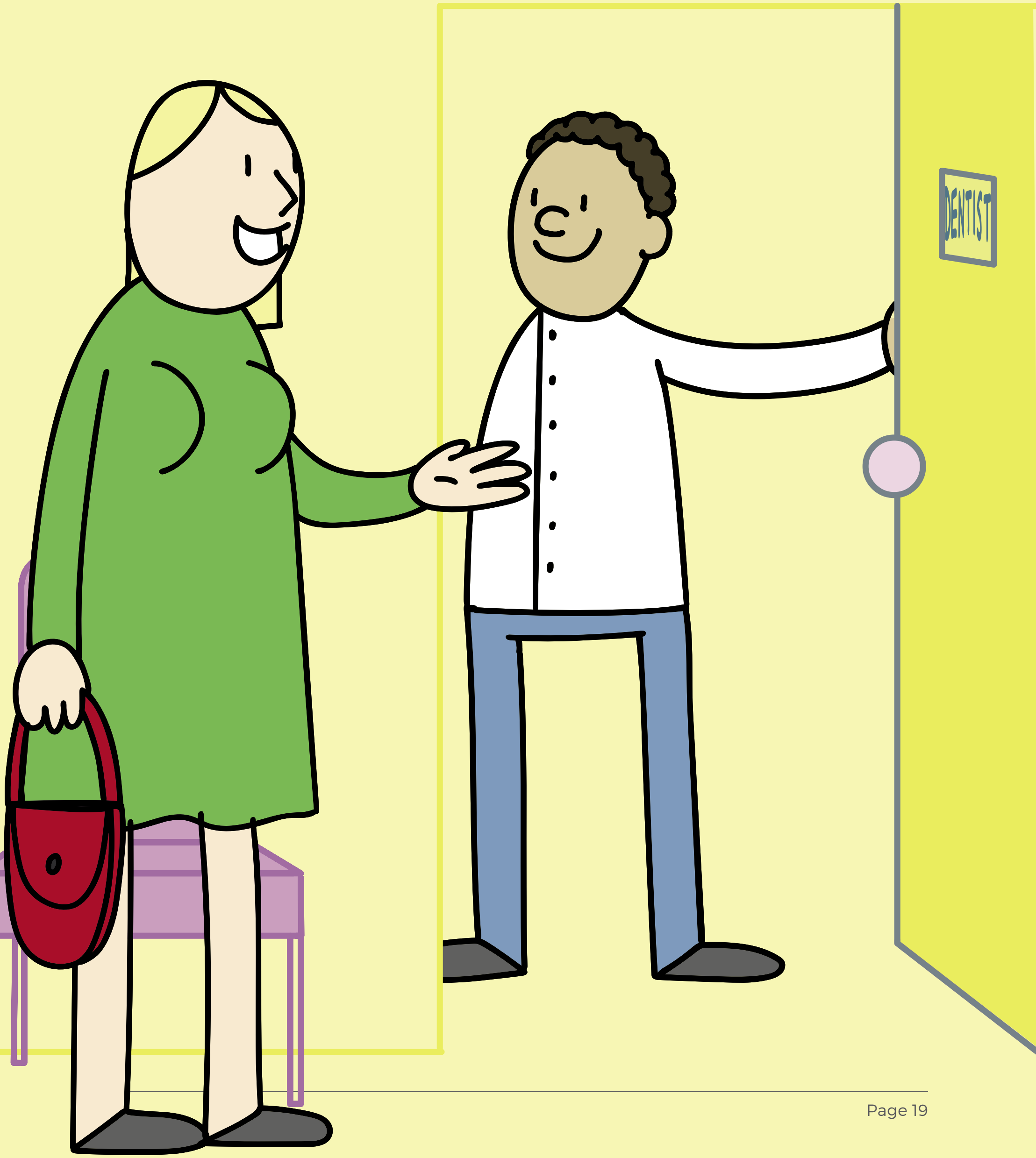

Supplement: Supplementary file 6 — Supplementary Material 6 [file 12875_2025_2732_MOESM6_ESM.pdf]
